# Characteristics of therapeutic alliance in musculoskeletal physiotherapy and occupational therapy practice: a scoping review of the literature

**DOI:** 10.1186/s12913-017-2311-3

**Published:** 2017-05-30

**Authors:** Folarin Babatunde, Joy MacDermid, Norma MacIntyre

**Affiliations:** 1 School of rehabilitation Science, McMaster University, 1400 Main Street West, Hamilton, ON L8S 1C7 Canada; 2 Hand and Upper Limb Centre, St Joseph Hospital, London, ON Canada; 3 Department of Physical Therapy, University of Western Ontario, London, ON Canada

**Keywords:** Therapeutic alliance, Musculoskeletal, Physiotherapy, Occupational therapy, Service delivery

## Abstract

**Background:**

Most conventional treatment for musculoskeletal conditions continue to show moderate effects, prompting calls for ways to increase effectiveness, including drawing from strategies used across other health conditions. Therapeutic alliance refers to the relational processes at play in treatment which can act in combination or independently of specific interventions. Current evidence guiding the use of therapeutic alliance in health care arises largely from psychotherapy and medicine literature. The objective of this review was to map out the available literature on therapeutic alliance conceptual frameworks, themes, measures and determinants in musculoskeletal rehabilitation across physiotherapy and occupational therapy disciplines.

**Methods:**

A scoping review of the literature published in English since inception to July 2015 was conducted using Medline, EMBASE, PsychINFO, PEDro, SportDISCUS, AMED, OTSeeker, AMED and the grey literature. A key search term strategy was employed using “physiotherapy”, “occupational therapy”, “therapeutic alliance”, and “musculoskeletal” to identify relevant studies. All searches were performed between December 2014 and July 2015 with an updated search on January 2017. Two investigators screened article title, abstract and full text review for articles meeting the inclusion criteria and extracted therapeutic alliance data and details of each study.

**Results:**

One hundred and thirty articles met the inclusion criteria including quantitative (33%), qualitative (39%), mixed methods (7%) and reviews and discussions (23%) and most data came from the USA (23%). Randomized trials and systematic reviews were 4.6 and 2.3% respectively. Low back pain condition (22%) and primary care (30.7%) were the most reported condition and setting respectively. One theory, 9 frameworks, 26 models, 8 themes and 42 subthemes of therapeutic alliance were identified. Twenty-six measures were identified; the Working Alliance Inventory (WAI) was the most utilized measure (13%). Most of the therapeutic alliance themes extracted were from patient perspectives. The relationship between adherence and therapeutic alliance was examined by 26 articles of which 57% showed some correlation between therapeutic alliance and adherence. Age moderated the relationship between therapeutic alliance and adherence with younger individuals and an autonomy support environment reporting improved adherence. Prioritized goals, autonomy support and motivation were facilitators of therapeutic alliance.

**Conclusion:**

Therapeutic Alliance has been studied in a limited extent in the rehabilitation literature with conflicting frameworks and findings. Potential benefits described for enhancing therapeutic alliance might include better exercise adherence. Several knowledge gaps have been identified with a potential for generating future research priorities for therapeutic alliance in musculoskeletal rehabilitation.

**Electronic supplementary material:**

The online version of this article (doi:10.1186/s12913-017-2311-3) contains supplementary material, which is available to authorized users.

## Background

Conventional treatments such as exercise commonly used in the management of musculoskeletal (MSK) conditions continue to show only moderate effects [1–3]. Research aimed at improving the effectiveness of treatment for MSK conditions should extend beyond condition specific interventions to include more general mediators of treatment such as communication or psychological interactions between patients and clinicians. One aspect of this is therapeutic alliance (TA) which has been described as the working relationship or positive social connection between the patient and the therapist [4] and established between therapist and client through collaboration, communication, therapist empathy, and mutual respect [5]. TA is a central component of the therapeutic process and is a determinant of treatment outcome [6, 7]. The origin of TA dates to back to Freud’s theory of transference and countertransference [6]. According to Bordin [4], TA can be applied to all change situations independent of the treatment modality and proposed a tripartite model of TA [8] consisting of three essential elements: agreement on the goals of the treatment, agreement on the tasks, and the development of a personal bond (reciprocal positive feelings) between the client and therapist.

TA has been studied extensively across a range of psychotherapy treatment modalities and aetiologies [9, 10] with recent findings showing a correlation with satisfaction, quality of life [11], psychological well-being [12], and symptom improvement [7]. Studies in medicine show that TA influences chronic disease care [13], improves adherence, satisfaction and quality of life [14], enhances communication [15, 16] and impacts decision quality [17]. This is opposed to recent interest in allied health disciplines like physiotherapy (PT) [20, 21] and occupational therapy (OT) [22]. Findings from physical rehabilitation show that TA is linked to engagement in stroke rehabilitation [16] and treatment outcomes in cardiac [17] and musculoskeletal (MSK) [18, 19] rehabilitation. It is notable that many studies used a TA conceptualization and outcome measures developed from psychotherapy and did not address TA as a primary research area. It also remains difficult to decide if outcomes are determined by specific techniques, mechanism of action or general processes like the TA [23]. This continues to limit the application of TA conceptualization from psychotherapy to PT and OT.

Furthermore, it remains unclear whether patient or therapist characteristics most determine outcome [24] and despite similarities, patient and therapist views of the key factors for effective TA may differ in important ways [25]. For example, it has been reported that clients view the TA in terms of collaborative work relationship, active commitment, bond, productive work, confident progress and agreement on goals/tasks while therapists focused on therapist confidence and dedication, client commitment and confidence, client working ability, and collaborative work relationship [25]. Thus, clients place greater emphasis on helpfulness, joint participation in therapy and negative signs of TA compared to therapists. Adherence is a patient characteristic linked to therapeutic change and considered an area of priority in MSK research and practice [26–28]. In physical rehabilitation, adherence has the potential to unlock some of the problems associated with understanding how TA exert its effect. Recent evidence shows TA may be the best predictor of adherence to exercise in MSK PT practice [29] and facilitator of patient engagement in OT practice [30]. Identifying the components of therapy responsible for symptomatic change would aid in the theoretical understanding of the processes underlying therapeutic change, improve practice and support development of effective practice [31]. Delineating the role of TA as a mediator, predictor or moderator of adherence may enhance understanding of TA as a therapeutic agent in MSK practice [32].

Based on these shortcomings in the TA literature, a comprehensive review of primary research in TA is required to map the breadth of literature for MSK conditions to advance knowledge in the following areas: conceptualization, active ingredients, psychometrically sound measures, mechanism of effect, and the mediating, moderating or predicting effect of TA on adherence. To this end, we conducted a scoping review of TA in MSK practice informed by the disciplines of OT and PT. The purpose of the study was to describe the type of research conducted to investigate the relationship between TA and rehabilitation of MSK conditions. Specifically, this review intends to describe to what extent the literature has theoretical underpinnings or a common understanding of what constitutes TA, has addressed the relationship between TA and adherence to treatment or treatment outcomes and how TA is measured.

## Methods

This scoping review was informed by Levac et al. [33] and Arksey and O’Malley [34] methodology. Scoping reviews are used to answer broad questions, synthesise information from a heterogeneous data pool or assess whether the literature is amenable to systematic review [35, 36]. This review employed the five-stage framework as outlined by Arksey and O’Malley: 1) identifying the research question, 2) identifying relevant studies, 3) selecting the studies, 4) charting the data (data extraction), and 5) collating, summarising and reporting the results. Reporting the results includes the use of numerical summaries that describe study characteristics. Levac et al’s recommendations focus on clarifying and enhancing each stage of the framework as follows: (stage 1) expounding and linking the research purpose and question; (stage 2) harmonizing feasibility, breadth and comprehensiveness of the scoping process; (stages 3 and 4) using an iterative team approach for study selection and data extraction; (stage 5) integrating a numerical summary and qualitative thematic analysis, reporting results, and considering the implications of findings to policy, practice, or research; and (stage 6) incorporating a knowledge translation strategy though consultation with stakeholders.

### Identifying relevant articles

In consultation with a librarian, a search strategy was developed to identify publications addressing TA. The evidence was searched using electronic databases, references lists, and by hand searching key journals. Literature search for *physiotherapy* or *occupational therapy* were completed to identify experimental studies that discussed or investigated the relationship between TA and adherence to exercise in the management of adults with MSK conditions. Using a combination of key words and medical subject (MeSH) terms (Table 1) related to TA, eight databases were searched: MEDLINE, PsychINFO, Embase, AMED, SportDISCUS, REHABDATA, PEDro and OTseeker. The search strategy was customized to each database. A manual search of the reference lists of identified articles was also conducted. A sample search strategy for the search is outlined in Table 1. All searches were performed between July 2015 and September 2015 using a combination of search terms (Table 1). An updated search was done in January 2017.

**Table 1:** Sample search Terms and Search Strategy

Therapeutic Alliance	Adherence	Rehabilitation	Musculoskeletal Diseases (MSK)
Key words	Key words	Key words	Key words
[1] Therapeutic alliance (MeSH exploded not focused) (keyword)	[12] Adherence (MeSH exploded not focused) (keyword)	[22] Rehabilitation (explode not focused) (keyword)	[29] Musculoskeletal diseases (MESH exploded not focuses) (keyword)
[2] Patient therapist relationship	[13] Adhere^a^ (keyword)	[23] Physiotherapy^a^	[30] MSK (keyword)
	[14] compliance	[24] Physical therapy^a^	[31] List diseases if you like
[3] Working alliance	[15] behaviour	[25] Exercise	Combine 29 or 30 or….. ➔ 31
[4] Therapeutic relationship	[16] behavior	[26] Exercise therapy	
	[17] concordance	Combine 22 or 23 or 24 or 25 or 26 ➔ 27	^a^Combine 28 and 31
[5] Collaboration	Combine 12 or 13 or 14 or 15 or 16 or 17 ➔ 18		^a^Add limitations (Inception to May, 2015, Adults aged 18 and above, humans, English Language, real patient and therapists)
[6] Helping alliance		28. Combine 21 and 27	
[7] Patient acceptance of health care (descriptors)	19. Combine 11 and 18		
	20. Adherence – MeSH + Group 2 MeSH		
[8] Attitude			
[9] Bond	21. Combine 19 or 20		
[10] Collaboration			
Combine 1 or 2 or 3 or 4 or 5 or 6 or 7 or 8 or 9 or 10➔ 11			

^a^This was substituted with occupational therapy or vocational rehabilitation in a separate search

### Study selection

After the initial search was completed abstracts and titles from the database searches were screened for relevance by the first reviewer (F.B.) and selected if they met the following criteria: [1] quantitative, qualitative or mixed methods data in a peer-reviewed journal, [2] systematic reviews and meta-synthesis, [3] experiences and/or perspectives of the therapist, observer and/or patient, [4] highlight TA or an aspect of TA as the main conceptual focus of the article, [5] findings relevant to MSK rehabilitation from adult population, [6] English language articles. Studies were excluded if they reported mixed population data without clearly highlighting MSK conditions or involved surgical and medical interventions alone. All references were imported to EndNote X7 software© and all duplicates deleted. Full texts of potentially relevant articles were retrieved and scrutinized by the first author (F.B.) and second author (J.M.) for consensus before final inclusion in the study.

### Data abstraction

Data related to TA were extracted from articles meeting the inclusion criteria by the lead author (FB) and reviewed by a second author (JM). Each article was first categorized based on study methods (quantitative, qualitative, mixed-methods, systematic review or meta-synthesis, narrative reviews or discussion paper) and level of care (primary, secondary, rehabilitation, private, community, home care, long term care). The following information was extracted and synthesised in summary format from the articles: authorship, publication year, country, setting, discipline, aims, design, participants, themes, and findings. Secondary data extracted included information on the conceptualization of TA; frameworks, theories or models, relationship between TA and adherence to exercise, measures of TA and participant perspectives on TA themes.

### Analysis

Descriptive statistics were calculated to summarize the data. Frequencies and percentages were used to describe nominal data (study characteristics, themes, measures). A narrative synthesis approach [37, 38] facilitated the mapping of the core themes of TA that emerged from this review. We used a thematic analysis to gather information and identify all TA themes. Inductive analysis was adapted and followed 3 stages: 1) extracting findings and coding findings for each article, 2) grouping of findings (codes) according to the topical similarity to determine whether findings confirm, extend, or refute each other; and 3) abstraction of findings (analyse grouped findings to identify additional patterns, overlaps, comparisons, and redundancies to form a set of concise statements that capture the content of findings).

## Results

**Fig. 1:**
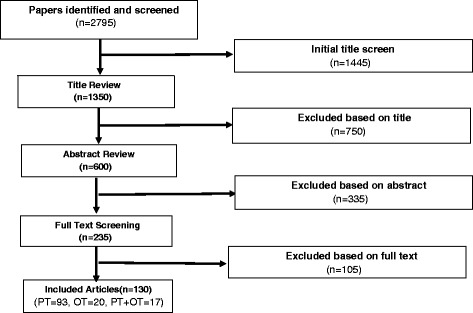
Search and screen of articles flow chart

The initial literature search of the TA literature resulted in an identification of 2795 titles. Of these titles, 691 were duplicates, 189 were book titles, 24 were non-English language articles and 482 did include an exercise or physical activity component. An additional 1279 were removed because they did not meet the eligibility criteria: letters, commentaries, editorials or conference abstracts (*n* = 426), titles from nursing (*n* = 160) and medicine (*n* = 263) and titles from psychotherapy (*n* = 430) literature. After final abstract and full text screening, 130 articles were selected as listed in (1 s–130 s) (See Additional file 1). The flow of articles through the review is shown in Fig. 1.

### Study characteristics

**Fig. 2:**
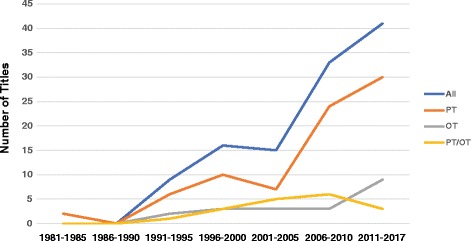
Number of studies published in each 5-year period from 1981 to 2016

Participants included 7,018 patients, 1225 OTs and 994 PTs. By country, most of the publications originated from the USA (23%) or Australia (16.9%). By continent, Europe accounted for 44.6% of the studies as compared to 30% from North America and 23% from Australasia. There was only one study published in South America, only 2 studies from Asia and none from Africa. The earliest study dates to a 1981 with an increase in publications (*n* = 46) between 2011 and 2016 and most of the studies originated from the PT discipline as depicted in Fig. 2. The most reported settings were primary care (34%) and outpatients (25%). Spinal (25%) and degenerative joint (21%) conditions were the most reported health condition studied. In some cases (19%) the details of the condition treated were not reported. All but two of the experimental studies were from the PT literature (Additional file 1).

### Study design

Overall, there were more quantitative (*n* = 43, 33%) than qualitative studies (*n* = 51, 39%) as shown in Table 2. Mixed method studies accounted for 7% and the remainder were narrative reviews and opinion papers (23%). Amongst PT studies there were similar amounts of quantitative and qualitative research; 77% versus 80% respectively. Experimental studies represented 4.6% of the eligible studies as compared to correlational (9.2%) and descriptive (17%) studies. Survey studies represented 9.2% and measurement studies was 5.3%. There was varied qualitative study methodologies including grounded theory (10%), phenomenology (16.9%), narratives (6.1%), ethnography (1.5%), case studies (2.3%), nominal group technique (1.5%) and symbolic interactionism (0.7%). Semi-structured interviews (17.6%) and focus groups (7.7%) were the most reported data collection techniques.

**Table 2:** Study characteristics

Characteristics	Frequency	*PT*	*OT*	*PT* + *OT*
	N (130) (%)	(*N* = *92*)	(*N* = *21*)	(*N* = *17*)
*Source country of study*				
Australia	22 (16.9)	18	2	2
Brazil	1 (0.7)	1	-	-
Canada	8 (6.2)	6	2	-
Germany	3 (2)	2	-	1
Hong Kong	2 (1.5)	2	-	-
Iceland	2 (1.5)	1	1	-
Ireland	2 (1.5)	2	-	-
Netherland	4 (3.1)	4	-	-
New Zealand	8 (6.2)	6	-	2
Norway	2 (1.5)	2	-	-
Spain	3 (2.3)	3	-	-
Switzerland	2 (1.5)	1	-	1
Sweden	12 (9.2)	8	3	1
Turkey	1 (0.7))	1	-	-
United Kingdom	27 (20.8)	23	2	2
USA	31 (23.8)	12	11	8
*Study design*				
Quantitative methods	43 (33.1)	32	6	5
Qualitative methods	51 (39.2)	41	8	2
Mixed methods	8 (6.2)	4	2	2
Narrative review articles/discussion papers	24 (18.5)	11	5	8
Systematic reviews	4 (3.1)	4	-	-
*Setting*				
Community	11 (10)	10	1	-
Long term care	1 (3)	-	1	-
Outpatient	25 (21)	19	3	1
Occupational Health	3 (4)	3	-	1
Primary care	41 (34)	33	5	3
Private practice	17 (18)	14	3	-
Rehabilitation	15 (10)	8	5	2
Various (multiple settings)	7 (6)	4	3	-
*Conditions*				
Spinal disorders	33 (25.3)	32	-	1
Degenerative disease	27 (20.7)	22	-	2
General Various	19 (14.6)	17	-	2
Wrist/Hand	10 (7.6)	10	-	-
Traumatic injury	9 (6.9)	8	1	-
Upper Limb	15 (11.5)	12	2	1
Workplace injury	2 (1.5)	2	-	-

### Conceptualization of therapeutic alliance

Several theories, models and frameworks related to TA were identified from 16 studies in the literature as shown in Table 3. There were 18 models represented in the literature including model of human occupation, health belief model, health locus of control, ecological model of adherence, transtheoretical model, self-regulation model, tripartite efficacy model, process model of collaboration, biopsychosocial model, consumer model, model of empathic understanding, resource conservation model, self-management model, model of helping encounter, Information-Motivation-Behavioral model, Gelso and Cater model, model of physiotherapist-patient interactions and independent living model. Eleven frameworks were reported and represented concepts from health behaviour change, compliance, illness perception, self-efficacy, patient beliefs, patient-centred care, satisfaction, helping, and partnership (Table 3). Three theories were linked to TA (Table 3) including self-determination theory (SDT), self-efficacy theory and social learning theory. SDT was the only theory with reported empirical evidence of effectiveness for promoting therapists’ supportive behavior during clinical practice in MSK PT practice [63].

**Table 3:** Conceptualization of therapeutic alliance

Author	Context	Origin	Conceptualization	Description	Therapeutic Alliance Themes
Chan et al., 2009 (10s)	^a^Whitlock et al. 5A’s framework of behaviour change	Existing literature on the self-determined motivation and engagement in health-promoting behavior.	^b^Self-determination theory	A. CONNECT	1. Partnership
				Communication style and exercise compliance in physiotherapy	2. Congruence
Levy et al., 2008 (30s)			^c^Physiotherapist Psychological		3. Communication
					4. Personalized therapy
Murray et al., 2015 (33 s)			Support		
				B. Ask, Advise, Agree, Assist, Arrange	
Chen et al., 1999 (13 s)	^a^Compliance and satisfaction with exercise	Existing literature and empirical study on compliance to home exercise in upper extremity rehabilitation	^c^Model of Human Occupation	1. Input	1. Communication
				2. Output	2. Connectedness
			^c^Health locus of control	3. Environment	3. Partnership
				4. The open system	4. Influencing factors
			^c^Health belief model		
Gorenberg et al., 2014 (109 s)	^a^Therapeutic use of self	Conceptual practice model for occupational therapy focused on understanding therapeutic use of self.	^c^The Intentional Relationship Model	1. Client	1. Connectedness
				2. Interpersonal events	2. Roles and responsibilities
				3. Practitioner	3. Partnership
				4. Occupational engagement	4. Congruence
Harman et al., 2012 (63 s)	^a^Building blocks of health behavior change	Existing literature on, empirical studies about behaviour change and low back pain rehabilitation	^c^Transtheoretical model	1. Need for action	1. Connectedness
				2. Solutions	2. Partnership
					3. Support
			^c^Motivational model of patient self-management	3. Reducing threat	4. Partnership
					5. Congruence
Hinman et al., 2015 (65 s)	^c^Model of Health Change	Existing literature on motivational interviewing, solution-focused coaching and cognitive behavioural therapy.	^c^Dimensions of health service delivery	1. Practice principles,	1. Connectedness
				2. Essential techniques	2. Congruence
				3. Step framework	
Hurley et al., 2007 (66 s)	^a^Understanding of illness	Parallel processing framework with one arm dedicated to cognitive processing of internal and external stimulus and the processing of emotional aspects of that stimulus.	^c^Levanthal’s self-regulation model of illness	1. Identity	1. Connectedness
				2. Timeline	2. Partnership
				3. Consequence	3. Influencing factors
				4. Cause	
				5. Control and cure	
				6. Illness coherence	
Jackson et al., 2012 (25 s)	^a^Tripartite efficacy framework in client-therapist rehabilitation interactions	Existing literature and empirical studies on efficacy beliefs	^c^Tripartite efficacy model	1. Client-related factors	1. Connectedness
					2. Role and responsibilities
				2. Therapist related factors	
			^b^Self-efficacy theory; relation-inferred self-efficacy		3. Personalized therapy
					4. Emotional support
					5. Communication
Jensen and Lorish, 1994 (26 s)	^a^Behavioral theory- based strategies for enhancing patient treatment cooperation and patient beliefs	Existing literature on compliance, decision-making, cognitive behavioral therapy and the explanatory model of exercise and mailed surveys to PTs	^c^Process Model of collaboration	1. Therapeutic relationship	1. Connectedness
				2. Problem solving	
				3. Negotiation	
				4. Mutual enquiry	
Kidd et al., 2011 (68 s)	^a^Patient centred care	Existing literature and empirical studies on patient-centred care	^c^Biopsychosocial model	1. Ability to communicate	1. Connectedness
				2. Understanding of people and ability to relate	2. Partnership
			^c^Patients perception of a good physiotherapist		
				3. Knowledge and expertise	3. Influencing factors
				4. Confidence	4. Communication
				5. Transparent focus on progress and outcome	5. Role and responsibility
Knight et al., 2010 (29 s)	^a^Client Satisfaction	Existing literature on satisfaction, and physiotherapy and empirical study on patient satisfaction.	^c^Consumer model	1. Service	1. connectedness
				2. Satisfaction	2. Influencing factors
				3. Dissatisfaction	3. Partnership
				4. Quality	4. Congruence
				5. Reasons for seeking therapy	5. Communication
Neuman et al., 2009 (116 s)	^c^Effect model of empathic communication in clinical encounter	Existing literature and hypothesis on clinical empathy	^c^Model of empathic understanding and adherence to treatment regimens (nature)	1. Cognitive action oriented effects	1. Communication
				2. Affective oriented effects	2. Partnership
Niederman et al., 2011 (34 s)	^a^Pictorial Representation of Illness and Self Measure	Existing literature on stress, coping strategies and resource utilization	^c^Hobfil’s resource conservation model	1. Self	1. Activating resources
			^b^Social learning theory	2. Resource	2. Treatment goals
				3. Separation	
			^c^Self management		
Norby and Bellner, 1994 (76 s)	^a^Dimensions of helping	Existing literature and empirical study on basic assumptions of occupational therapy	^c^Tentative model of the helping encounter	1. Basic Professional-Oriented helping	1. Connectedness
				2. Understanding-Oriented helping	2. Partnership
				3. Action-Oriented helping	3. Roles and responsibilities
Radomski, 2011 (118 s)	^c^Ecological model for adherence in rehabilitation	Existing literature on adherence and occupational therapy	^c^Transtheoretical model of change	1. Person factors	1. Congruence
				2. Provider factors	2. Connectedness
				3. Intervention factors	3. Communication
			^b^Self-determination theory		
				4. Technology	4. Influencing factors
				5. Social	
				6. Environmental	
Schoster et al., 2005 (100 s)	^c^Information	Existing literature and empirical studies on predicting HIV-preventive behaviour	^c^Information-Motivation-Behavioural skills model	1. Exercise information	1. Connectedness
	Motivation and Behavioural model				
				2. Exercise motivation	2. Roles and responsibilities
				3. Exercise behavioural skills	3. Influencing factors.
				4. Barriers	4. Partnership
				5. Exercise behaviour	
Szybek et al., 2000 (121 s)	^c^Model of Physiotherapist-patient interactions	Existing literature on Psycho-therapeutic encounters, working alliance, transference and real relationships	^c^Gelso and Carter model	1. Interactions	1. Partnership
				2. Non-insight oriented therapist	2. Congruence
				3. Insight oriented therapist	
Verkaaik et al., 2010 (123 s)	^a^Productive partnership (P2) framework	Existing literature on power distribution in partnerships	^c^Independent living movement model	1. Context	1. Partnership
				2. Predicted characteristics	
			^c^Consumer direction model	3. Autonomy	
				4. Knowledge	

^a^Frameworks (*n* = 10), ^b^Theories (*n* = 3), ^c^Models (*n* = 19)

### Therapeutic alliance themes

The initial coding of the 130 eligible articles resulted in 44 codes, which were reduced and organized into 8 themes: congruence, connectedness, communication, expectation, influencing factors, individualized therapy, partnership, and roles and responsibilities and described in (See Additional file 2). Table 4 shows that agreement on goals (32%) was the most reported aspect of congruence. Friendliness (21%) was the most reported characteristic of connectedness followed by a perception of a good relationship and genuine interest or concern at 14%. Clarity of information (26%), active listening (39%) and nonverbal skills (24%) were the most represented characteristics of communication. Expectation was approximately equally represented with regards to both therapy (25%) and outcome (22%). External factors (17%) and therapist skill and competence (30%) were the most identified influencing factors. Patient life experiences (11%) and willingness to engage (11%) were the most reported patient prerequisite. Being responsive and holistic practice were important to individualized therapy (14.6%). Mutual understanding (23%) and active involvement (28%) were the most important partnership characteristics. Therapist ability to activate patient resources (13.1%) and motivating or encouraging patients (26%) were the most reported role and responsibility.

**Table 4:** Core Themes of Therapeutic alliance

Themes (*n* = 8)	Codes (*n* = 44)	No of studies N (%)
Congruence	Agreement on goals	32 (24.6)
	Problem identification	19 (14.6)
	Agreement on tasks	27 (20.7)
Connectedness	Perceived good relationship	14 (8.7)
	Friendliness	21 (20.3)
	Empathy	16 (9.7)
	Caring	15 (15.5)
	Warmth	13 (10)
	Genuine interest/concern^a^	14 (10.7)
	Therapist faith/belief^a^ in patient	8 (7.7)
	Honesty^a^	2 (1.5)
	Courtesy^a^	4 (3.0)
Communication	Nonverbal	24 (18.4)
	Listening skills	39 (30)
	Visual aids	7 (5.3)
	Clear explanation and information^a^	26 (20)
	Positive feedback^a^	9 (6.9)
Expectation	Therapy	25 (19.2)
	Outcomes	22 (16.9)
Individualized therapy	Responsiveness	9 (6.9)
	Holistic practice	8 (7.7)
Influencing factors		
External factors	Structures, processes and environment	17 (13.1)
Therapist prerequisite	Skill and competence and experience	30 (23.1)
	Personal characteristics	13 (10)
	Humor	7 (5.3)
	Life experiences	7 (5.3)
	Emotional intelligence^a^	3 (2.3)
Patient prerequisite	Personal characteristics	6 (4.6)
	Existing resources	10 (7.7)
	Life experiences	11 (8.4)
	Willingness to engage	11 (8.4)
Partnership	Trust/dependability	23 (17.6)
	Respect	19 (14)
	Mutual understanding	24 (18.4)
	Knowledge exchange	19 (14.6)
	Power balance	6 (4.6)
	Active involvement/engagement	28 (21.5)
Roles and responsibilities	Activating patient’s resources	17 (13.1)
	Motivator/Encourager^a^	26 (20)
	Professional manner	13 (10)
	Educator/Adviser^a^/Guide^a^	11 (8.4)
	Active follow-up^a^	5 (3.8)
	Autonomy support^a^	3 (2.3)

^a^New component descriptors identified from in this review

### Therapeutic alliance outcome measures

Twenty-seven measures were identified in 37 studies as shown in Table 5. Six studies were from OT literature and 4 involved both OT and PT participants. Psychometric properties were reported for 21 measures (77%) from 28 studies. The Working Alliance Inventory (WAI) [39, 40] was the most utilized measure (5 studies) among available studies. Other alliance-type measures (3 studies) were the working subscale of the Pain Rehabilitation Expectation Scale (PRES) [41], the Helping Alliance Questionnaire (HAq) [42], communication preferences of patients with chronic illness questionnaire [43] and revised version of the Helping alliance questionnaire [44]. Four measures from 4 studies focused on satisfaction; Medical Interview Satisfaction Scale (MISS) [45], Health Care Satisfaction questionnaire [46], MedRisk [47] and Physiotherapy Outpatient Satisfaction questionnaire [48]. Three measures that focused on empathy: Consultation and Relational Empathy scale [49], Barett-Lennard Relationship scale [50] and Truax Accurate Empathy Scale [51]. One measure focused on communication; the Medical Communication Behaviour System [52]. Therapist support was the focus of 2 measures; the Health Care Climate Questionnaire (HCCQ) [53] (3 studies) and the Relationship Assessment Scale (RAS) (1 study). The Clinical Assessment of Modes [54] was used to assess therapeutic use of self in one study from OT discipline. The Patients’ Experiences in Postacute Outpatient Physical Therapy Settings [55] was the only measure developed specifically for a rehabilitation setting. The information about and content of each TA measure was also coded against the themes of TA identified in literature and the PRES [41] was the only measure reflecting all the eight TA themes. Ten measures (37%) reflected at least 5 TA themes.

**Table 5:** Measures of Therapeutic Alliance

Articles	Outcome Measure	Therapeutic Alliance Themes								Psychometrics
		C	Cm	E	I	P	Pt	Co	Rr	
Adamson et al., 1994 (1 s)	Attitude scale (19-item)				X	X	X	X	X	Yes^a^
Stenmar and Nordholm, 1994 (101 s)										
Baker et al., 2001 (4 s)	Participation Method Assessment Instrument (21-item)	X			X	X	X			Yes^b^
Beattie et al., 2005 (6 s)	MedRisk Instrument for Measuring Patient Satisfaction with Physical Therapy Care (MR-12) (12-item)		X		X	X		X		Yes^a^
Besley et al., 2010 (7 s)	Health Alliance Questionnaire (HAQ) (19-item)	X		X	X	X		X		Yes^a^
Bliss, 2010 (8 s)	Working Alliance Inventory (WAI-12) (12-item)	X			X	X				Yes^a^
Besley et al., 2010 (7 s)										
Burns et al., 1999 (9 s)										
Morrison, 2013 (98 s)										
Chan and Can, 2010 (10 s)	Self-developed questionnaire (5-item)		X			X				No
Cole and McLean, 2003 (14 s)	Self-developed questionnaire (10-item)	X	X			X		X	X	No
Eklund et al., 2015 (17 s)	Working Relationship Questionnaire (HAqII) (19-item)	X		X	X	X	X	X	X	Yes^a^
Farin et al., 2011 (57 s)	KOPRA questionnaire (32-item)	X	X			X		X		Yes^a^
Ferreira et al., 2013 (18 s)	Working Alliance Theory of Change Inventory (WATOCI) (16-item)	X		X		X	X	X		Yes^b^
Hall et al., 2012 (23 s)										
										Yes^a^
Cheing et al., 2010 (12 s)	Pain Rehabilitation Scale (PRES) (54-item)	X	X	X	X	X	X	X	X	Yes^a^
Fuentes et al., 2014 (20 s)										
Vong et al., 2011 (42 s)										
Gorenberg and Taylor, 2013 (109 s)	Clinical Assessment of 5 Modes Scale (23-item)	X				X	X	X	X	Yes^a^
Taylor et al., 2011 (39 s)										
Grannis et al., 1981 (22 s)	Q sort questionnaire (28-item)		X		X	X		X		No
Hills and Kitchen, 2007 (24 s)	Physiotherapy Outpatient Satisfaction Questionnaire (38-item)		X	X	X			X		Yes^a^
Jackson et al., 2012 (25 s)	Relationship Assessment Scale (RAS) (16-item)	X	X		X	X	X		X	Yes^a^
Kersten et al., 2012 (27 s)	Consultation and Relational Empathy (CARE) (10-item)		X			X	X	X		Yes^a^
Kerssens et al., 1999 (28 s)	Self-developed questionnaire (11-item)	X	X					X	X	No
Knight et al., 2010 (29 s)	Service dimension questionnaire (12-item)		X		X	X		X	X	Yes^a^
Lysack et al., 2005 (31 s)	Self-developed questionnaire (3-item)	X				X		X	X	No
Medina-Mirapeix et al., 2015 (32 s)	Patient Experience in Post-Acute Outpatient Physical Therapy (PEPAP-Q)		X	X	X	X	X	X	X	Yes^a^
Murray et al., 2015 (33 s)	Health Care Climate Questionnaire (HCCQ) (6-items)		X			X		X	X	Yes^a^
Chan et al., 2009 (10 s)										
Levy et al., 2008 (30 s)										
Roberts and Bucksey, 2007 (36 s)	Medical Communication Behaviour System (23-item)		X		X				X	Yes^a^
Roberts et al., 2013										
Thomson et al., 1997 (40 s)	Barrett-Lennard Relationship Inventory (BLRI) (64-item)	X				X		X	X	No
Thomson et al., 1997 (40 s)	Truax Accurate Empathy Scale (TAES) (8-item)							X		No
Tousignant, 2011 (41 s)	Health Care Satisfaction questionnaire (26-item)				X			X		Yes^a^
Sluijs et al., 1991 (37 s), 1993 (38 s)	Patient Education checklist (5-item)								X	Yes^a^
Wright et al., 2013 (43 s)	Medical Interview Satisfaction Scale (26-item)		X		X	X		X	X	Yes^a^

Key: C-Congruence, Co-connectedness, Cm-Communication, E-Expectation, I-Influencing factors, P-Partnership, Pt-Personalized Therapy, Rr-Roles and Responsibilities

^a^Psychometric property tested in study

^b^Psychometric property reported from another study

### Therapeutic alliance and treatment adherence

Twenty-six articles examined the relationship between TA and treatment adherence as summarized in Table 6. More quantitative studies (50%) examined adherence compared to qualitative (42%) and mixed method studies (7.6%). The WAI-12 [39], PRES [41], MISS [45] and HCCQ [53] were the validated TA measures reported in the literature when investigating the relationship between TA and adherence. The Sports Injury Rehabilitation Adherence Scale (SIRAS) [56] was the most reported exercise adherence measure. Two studies (7.6%) reported no change in adherence with enhanced TA compared to 3 studies (11.5%) where improvement in adherence was reported. Improved patient-therapist relationship accounted for 18–23% of the variance in patient adherence. Patients and therapists acknowledge that effective communication improved adherence. Therapists (PT) reported that pleasing the therapist, activating patient resources and connectedness, faith in the therapist and shared goals are reasons for improved adherence. In, one study patients reported enhanced TA was not important for improved adherence. However, in other studies (53.8%) patients, TA characteristics predictive of exercise adherence included agreement on goals and tasks, clear communication, sense of connectedness, positive feedback, boosted, genuine interest, individualized care, trust in therapist and feeling empowered are important for developing exercise adherence behavior. Moderators are “pre-randomized” baseline characteristics that interact with treatment to influence the direction or magnitude of outcomes [57]. Levy et al. [58] showed that age moderated the relationship between TA and clinic-based adherence with younger and more autonomous individuals being more adherent to treatment. Predictors are baseline characteristics that predict response in both treatment and control groups [59]. Mediators are variables responsible for all or parts of the effects of a treatment or outcomes. They change during treatment, are associated with treatment and must influence outcome to be considered a mediator [57]. In this scoping review, prioritization of goals, autonomy support and motivation mediated the relationship between TA and adherence.

**Table 6:** Relationship between therapeutic alliance and adherence to treatment

Studies	Aim	Population	Design	Therapeutic Alliance Measure	Adherence Measure	Results
Bliss, 2010 (8 s)	To examine psychosocial variables like attachment style, depression and the working alliance as predictors of treatment outcomes	Chronic knee pain (*n* = 59)	Correlational study	Working Alliance Inventory	5-item self-report measure of treatment compliance (α = 0.83)	The transformed WAI scores were significantly positively correlated to pain interference and severity, patient compliance and satisfaction. The transformed WAI accounted for 24% of the variance in patient compliance
Campbell et al., 2001 (48 s)	To understand reasons for compliance and non-compliance with a home-based exercise regimen	Knee osteoarthritis (*n* = 20)	Grounded theory with thematic analysis	Interviews	Interviews	Compliance were apparent initially when attending PT sessions and later when a number of factors combined to determine continued and long term compliance (or non-compliance). Continued compliance depends on a person’s perception of their symptoms, the effectiveness of the intervention, their ability to incorporate it into everyday life and support from physiotherapists.
						A model of continued compliance was developed.
Chan and Can 2010 (11 s)	To evaluate patients’ adherence to home exercise programs in clinical practice and understand factors that affect patients’ adherence to home exercises.	Orthopaedic, sports injury, hand therapy, rheumatology (*n* = 82)	Cross-sectional survey study	25 item questionnaire	5-item exercise performance questionnaire.	Motivation, role of exercise, patients’ understanding of exercises, verbal and visual explanation and satisfaction with PT were found to have a strong effect on patient’s performance of home exercises.
Chan et al., 2009 (11 s)	To investigate the impact of PT’s autonomy-supportive behaviors on patients’ motivation and rehabilitation adherence	Anterior cruciate ligament injury (*n* = 115)	Correlational study	Healthcare Climate Questionnaire 15-item	Sport Injury Rehabilitation Adherence Scale (SIRAS)	Autonomous treatment motivation was associated positively with autonomy support but the relationship between autonomy support and controlled treatment motivation was not significant. Autonomous treatment motivation fully mediated the effect of physiotherapists’ autonomy-supportive behaviours on patients’ adherence.
					Patient self-report home-based exercise adherence	
Crook et al., 1998 (15 s)	To report the problem experienced with patient engagement in PT-led groups undertaking either an aerobic exercise or a stretching and relaxation program.	MSK disorders (*n* = 228)	Mixed methods study (quasi randomized controlled trial, interviews, checklist)	Individual interviews	Home exercise diary for exercise activity	PTs and patients acknowledge that listening was an importance part of the therapeutic relationship that improved adherence.
		LBP [52], Neck pain [30];				
		Lower limb pain [25], Shoulder pain [12]				
Escolar-Reina et al., 2010 (56 s)	To explore perceptions of people pain about the characteristics of home exercise programs and care-provider style during clinical encounters may affect adherence to exercises.	Chronic neck or low back pain (*n* = 34)	Grounded theory approach	Interviews	NA	Patient adherence to home-based exercise is more likely to happen when care providers’ style (clinical knowledge, feedback, giving reminders, monitoring adherence and promoting exercise feedback and the content of exercise programme) are positively experienced.
Freene et al., 2014 (96 s)	To compare a PT-led home-based PA program to usual practice of community group exercise program to determine effectiveness in middle-aged adults for increasing physical activity levels over the short and long term.	Sedentary community dwelling adults (*n* = 37)	Mixed methods study (quasi randomized trial, focus groups)	Interviews	Self-report on Active Australia Survey	Most participants agreed the physiotherapist was an enabling factor for the home-based intervention, although others did not think this was important. Participants reported a good interaction with the PT and felt they were expert and knowledgeable.
					Reliable and valid national measure.	
						Advice and support and individually tailored program from the PT and a good relationship with the instructor was important for continued participation in physical activity at home.
Gleeson et al., 1991 (21 s)	To develop policies and procedures about management of patient non-attendance in OT.	Hand injuries, burns, rheumatology (*n* = 100)	Cross-sectional survey study	Survey instrument	Patient and therapist comment on non-adherence	28% of patients believed that poor communication with the therapist was the reason for non-adherence.
						PTs felt that non- attendance affected continuity of care due to difficulty in evaluating the overall effectiveness of treatment, unmet goals, inability to establish ongoing plans, and concern regarding discharge. PTs saw non-compliance as the result of a need to develop personal skills (empathy, warmth, concern), demonstrating a feeling of responsibility for non-attendance.
Harman et al., 2012 (63 s)	To describe the approach used by a PT during a rehab programme for injured members of the military designed to enhance self-efficacy and self-management skills.	Chronic low back pain (*n* = 12)	Qualitative study with interpretive paradigm	Interviews	NA	Trusting the physiotherapist helped patients continue with their programme despite it getting harder, challenging their confidence, and not showing immediate results.
Hinman et al., 2015 (65 s)	To explore how patients, PTs and telephone coaches experienced, and made sense of an integrated program of PT-supervised exercise and telephone coaching.	Knee osteoarthritis (*n* = 6)	Grounded theory with symbolic interactionism	Interviews	Interviews	Patients felt accountable and responsible for meeting goals when perceived attention from PT was individualized and genuine.
						PTs appreciated providing clear information and monitoring progress, incorporation of exercise into daily routine. PTs recognized that collaboration, mutual understanding and emphasising the same treatment with the client as the central character were important.
Hurley et al., 2010 (66 s)	To explore the health beliefs, experiences, treatment expectations of people with chronic knee pain, and investigate if, how and why these change after taking part on an integrated exercise-based rehabilitation programme	Chronic knee pain (*n* = 29	Grounded Theory with thematic analysis	Interview	Attendance	The care, support and guidance participants received during the informal discussions helped build a trusting, collaborative partnership between patient and PT. This increased participant’ confidence and trust in the PT and belief in the rehabilitation programme. The interpersonal qualities and professional skills of the supervising PT were considered as important to the success of the programme as the content of the programme itself.
Jackson et al., 2012 (25 s)	To (i). explore potential relationship s between clients’ “tripartite” efficacy constructs, relationship quality with the therapist, and engagement in exercise and, (ii) model actor and partner effects or clients’ and therapists’ efficacy beliefs in relation to relationship quality	Osteoarthritis, osteoporosis, bursitis (*n* = 68)	Descriptive and Correlational study.	5-items from the 7-item Relation-ship adherence scale	3-item Engagement instrument	Increase in perception of relationship quality were directly related to improvements in engagement scores, accounting for 18% of the variance in engagement ratings.
Jensen et al., 1994 (26 s)	To integrate concepts from research, theory, and practice are integrated into a Process Model for Patient-Practitioner Collaboration for use in clinical practice	Rheumatoid arthritis.	Correlational survey study	Interview	NA	Pleasing the therapist was a reason for adherence to exercises prescribed.
		Osteoarthritis, low back pain (*n* = 305)				
		PTs (*n* = 568).				
Karnad and McLean, 2011 (67 s)	To explore PT’s perception of exercise adherence and interventions used in clinical practice.	Chronic MSK conditions	Interpretative Phenomenology	Interviews	Interviews	Most PTs believe that clear communication, faith in the PT, realistic treatment plans, shared goals and pain education are important for adhering to exercise.
		PTS (*n* = 5)				
Kingston et al., 2014 (97 s)	To determine whether compliance and understanding of a home exercise program is improved when patients are provided with a DVD.	Traumatic hand injury (*n* = 53)	Randomized controlled trial	Follow up survey	Compliance measures; diary recording of exercise, checklist for correctness and understanding of exercises, weekly attendance	No significant differences were found in the overall mean exercise compliance score between the groups.
						All participants reported that the instructions provided were easy to use (100%). All respondents (100%) felt that their appointment with their hand therapist was moderately to extremely important and 90.6% felt their appointment was moderate to extremely important in motivating them to do theirexercises.
Levy et al., 2008 (30 s)	To investigate the relationship between perceived autonomy support, age, and rehabilitation adherence among sports-related injuries	Tendon related injuries ankle, knee, shoulder, elbow) (*n* = 70)	Prospective correlational study	Healthcare Climate Questionnaire 15-item	Sport Injury Rehabilitation Adherence Scale (SIRAS)	High autonomy support provided by the physical therapist was related to better clinic-based adherence and attendance but not to home-based adherence. Age was related to all adherence indices and moderated the relationship between perceived autonomy support and clinic-based rehabilitation adherence.
					Clinic attendance	
					Home exercise adherence	
Liddle et al., 2007 (71 s)	To explore the experiences, opinions and treatment expectations of chronic low back pain patients to identify what components of treatment they consider as being of most value.	Chronic low back pain (*n* = 18)	Narrative study using focus group	Interviews	NA	Lack of faith in practitioner resulted in participants ignoring advice and failing to adhere to home exercises programs and continuing bad postural habits. Follow-up support and reassurance about correct exercise instructions and assistance with appropriate treatment progression improved exercise adherence.
Littlewood et al., 2014 (72 s)	To increase knowledge and understanding of the experience of exercising and determine perception of facilitators and barriers to exercise.	Rotator cuff tendinopathy (*n* = 6)	Phenomenology with framework analysis	Interviews	NA	PTs and patients agreed that ongoing support in the form of providing feedback, proactive follow-up and stimulating further engagement with the self-managed exercise programs when progress was slow were influential on successful outcomes
Lysack et al., 2005 (31 s)	To compare computer-assisted video instruction and routine rehabilitation practice on compliance and satisfaction with home exercise.	Total joint arthroplasty (*n* = 40)	Randomized controlled trial	3-item tool on encouragement, courtesy, and, active involvement	Self-report on exercise performance accuracy, difficulty in remembering exercises, exercise frequency, level of exercise when feeling poorly, and duration of each exercise session	Statistical analysis showed there were no significant differences at follow-up between the video and control groups on any of the exercise compliance items or on any of the patient satisfaction items (p > 0.05 in all cases). Results of this randomized trial suggest that computerized patient education technology may not provide the benefits anticipated.
		Hip [21]				
		Knee [19]				
					Rating of quality of exercise performance	
Petursdottir et al., 2010 (81 s)	To increase knowledge and understanding of the experience of exercising among individuals with osteoarthritis and to determine what they perceive as facilitators and barriers to exercising.	Osteoarthritis (*n* = 12)	Phenomenology	Facilitator and barrier checklist	NA	Many participants placed emphasis on the fact that the encouragement and understanding they received from their PT were very important.
		Hip/knee (*n* = 10)				
		Vertebral column (*n* = 9)				
						Clear communication and a sense of a positive connection were equally as important as the physical results of the therapy and adherence to exercise in physical therapy. Supervision by the PT facilitated exercise maintenance.
		Hands [6]				
		Other joints [3]				
Slade et al., 2009 (86 s)	To understand the factors that participants in exercise programs perceive to be important to engage and participate	Chronic low back pain (*n* = 18)	Grounded theory with focus groups	Audio-taped interviews	Audio-taped interviews	Helpful and empowering care-provider skills are those of the effective educator, motivator and communicator. Care-seekers are empowered by recognition of their own physical capability, motivators, time-management skills, and assertiveness to adhere to exercise
Sluijs et al., 1993 (38 s)	To investigate whether patent compliance was related to characteristics of the patient’s illness, attitude or physical therapist’s behaviour.	Trauma and postoperative conditions, Radiating back pain, Non-radiating back pain, Neck and shoulder pain (*n* = 1837)	Correlation study	5-item questionnaire	1-item questionnaire	The 5 forms of PT behavior showed no direct, statistically significant relationship with compliance.
						Compliance was significantly related to the positive feedback (therapist satisfaction with and appreciation of exercise performance).
		PT (*n* = 300)				
		Observers (*n* = 3)				
Stenmar et al., 1994 (101 s)	To find out the kinds of attributions PTs make regarding why PT works and the extent to which attributions are related to background variables.	PTs (*n* = 140)	Cross-sectional survey study	22 Likert-type items and various demographic variables.	NA	Majority of the respondents believed that the patient’s own resources and the patient-PT relationship rather than the treatment techniques are the most important factors in explaining why PT works. Other background factors had no relationship to the beliefs and attitudes expressed.
Veenhof et al., 2006 (91 s)	To understand why patients who have received a behavioural graded activity program successfully integrate activities into their daily lives.	Osteoarthritis (*n* = 12)	Grounded theory approach	Interview	Self-report on integrating activities into daily life after discharge	Initial motivation, active involvement in the whole process and that the PT coaching role during intervention facilitated adherence to exercises and activities:
Vong et al., 2011 (42 s)	To examine whether the addition of motivational enhancement therapy (MET) to conventional PT produces better outcomes than PT alone	Chronic low back pain (*n* = 76)	Randomized, controlled trial	Pain Rehabilitation Expectation Scale (PRES)	Exercise log (frequency)	The MET-plus-PT group produced significantly greater improvements than the PT group in proxy efficacy, working alliance, and treatment expectancy with significantly better performance in lifting capacity, general health and exercise compliance.
Wright et al., 2013 (43 s)	To identify which factors best explain non-adherence to home rehabilitation for patients with musculoskeletal injuries.	Musculoskeletal injuries (*n* = 87)	Cross-sectional study	Medical Interview Satisfaction Scale (MISS)	Sports Injury Rehabilitation Scale (SIRAS)	Patients are most likely to adhere to HRE when they perceive a positive relationship with their PT. Self-reported adherence is higher when patient perception of behavioural, cognitive and affective elements of the relationship are positive.

### Participant perspectives on therapeutic alliance

**Fig. 3:**
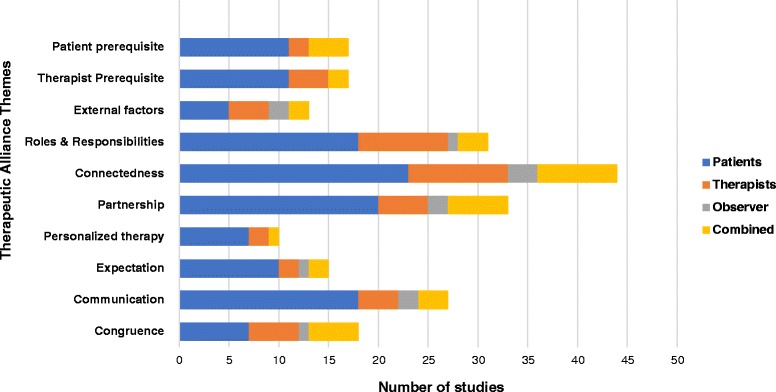
Perspectives of participants (patients, therapists, observers) on each theme of therapeutic alliance

To better delineate the phenomenon of TA, we analyzed the perspectives of TA among patients, therapists or observers as shown in Fig. 3 in the interventional and non-interventional studies. Observers were mostly researchers or other therapists not directly involved in patient care. Overall, most of the views shared were from patients. Twenty articles (15.3%) represented views on congruence out of which 9 reflected patient’s perspectives. Thirty-five articles (26%) represented views on communication with patient perspectives alone accounting for 65% of the articles. Sixteen articles (12%) reported perspectives on expectations with patient views representing 75%. Eleven articles (8%) represented views on individualized therapy out of which 7 studies portrayed patient perspectives. Thirty-nine articles (30%) represented views on partnership with patients’ perspective accounting for nearly half of all the articles. Fifty-two articles (40%) represented views on connectedness of which 28 articles represented patient views alone. Thirty-three articles (25%) identified the therapist role and responsibilities as key determinants of TA with patient perspective accounting for more than half of the data. Fifty-one articles (39%) represented participant views on influencing factors; patient prerequisites (37%), therapist prerequisites (35%) and external factors (27%). Among the 8 themes, communication, interpersonal aspects, partnership and roles and responsibilities were regarded as the most important determinants of TA according to patients. A breakdown of the code (subcategory) under each theme is highlighted in (See Additional file 3).

### Secondary synthesis of systematic reviews

There were 3 articles in the PT literature focused on reviewing the evidence on TA in this scoping review. There were 2 systematic reviews with a total of 11 studies. Besley et al. [60] examined a wide range of PT clinical practice but included 4 MSK studies and reported that there were 8 core components of TA including patient expectations, personalized therapy, partnership, therapist roles and responsibilities, congruence, communication, relational aspects, and influencing factors. The authors reported that the WAI [39], [40] and HAq [42] measures of alliance did not adequately address all the components of TA. The study by Hall et al. [61] included 6 MSK studies and reported 3 key components of TA including agreement in goals, agreement on interventions and affective bond. Three outcome measures of TA; WAI-12 [39], WAI-36 [40] and MedRisk [47] were used in the MSK studies. Hall et al. [61] reported positive associations between TA and global perceived effect, change in pain, physical function, patient satisfaction, depression and general health status. In a recent meta-synthesis of qualitative studies, O’Keeffe et al. [62] identified 4 themes of TA from 12 codes across 13 MSK studies. These included physical therapist interpersonal and communication skills (listening, empathy, friendliness, encouragement, confidence, nonverbal communication), physical therapist practical skills (patient education, physical therapist expertise and training), individualized patient-centred care (individualized, taking patient preferences and opinion into consideration) and organizational and environmental factors (time, flexibility with patient appointment and treatment).

## Discussion

This study represents a mapping of the breadth of the evidence for TA in PT and OT MSK practice and identified eight themes of therapeutic alliance valued by patients across different MSK settings and populations. Kayes and McPherson [63] identified that TA is increasingly regarded as an important determinant of engagement in physical rehabilitation but several gaps exists which hinder understanding of TA. This scoping review is an attempt to provide a foundation for future research by collating and summarizing the theoretical and empirical evidence concerning the construct “therapeutic alliance”, how it is currently measured and its relationship to adherence in MSK practice. This cataloguing of the evidence will assist in defining research questions and applying methodology that enables quality appraisal which is not a component of scoping review methodology [33]. The accord around characteristics of partnership, personalized therapy, roles and responsibilities, congruence, communication, expectations and influencers across PT and OT literature for MSK conditions identified in this scoping review provides further credence that these key themes should be included and evaluated in future studies or in clinical training. The synthesis findings mirror those of the systematic reviews by Besley et a [60] exploring TA in PT literature but this current study further expanded the key qualities linked to each theme. For example, our findings revealed several new subcategories such as humour and emotional intelligence (therapist prerequisites), appreciation, honesty (connectedness), clarity of information and feedback (communication), support and follow up (roles and responsibilities).

Various models and frameworks with diverse origins have been introduced to explain TA in PT and OT literature. The productive partnership framework [64] is based on power balance, the process model for patient-practitioner collaboration is based on shared-decision making [65], effect model of empathic communication [66] is based on connectedness and tripartite efficacy framework [67] is based on self-efficacy. Moreover, most of this conceptualization are yet to be empirically tested in the MSK population. The tripartite efficacy framework [67] opines that patients and therapists develop a “tripartite” network of efficacy beliefs. Although, the framework explains the motivational and relational processes for improving TA between patient and therapist during therapy encounters, it remains untested in MSK PT practice. The models of TA also had diverse origins ranging from traditional healthcare quality principles such as patient-centred care [68, 69] and important healthcare outcomes such as patient satisfaction [70] to modern models of emotion management such as emotional intelligence [71, 72]. This heterogeneity limits the application of this conceptualizations to broad MSK settings and conditions.

The construct of TA proposed by Bordin [8] is steeped in psychotherapy [73, 74] and viewed as a “pan-theoretical” concept of TA due to its applicability to many therapeutic approaches [75]. The question remains as to whether Bordin’s construct of TA is truly transferable to MSK rehabilitation. Findings from this scoping review highlights the importance of other constructs such as external influencing factors in establishing patient-therapist relationship in MSK practice. Praestegaard and Gard [67] reported that patients in private PT practice were not open to questions about their lived lives and therapists expressed difficulty in gaining important knowledge about their meaningful lives due to the impact of organizational factors such as available treatment time and design of treatment areas. Besley et al. [60] and O’Keefe et al. [61] also identified the environment as a significant influence on TA in the studies on MSK PT practice. Individuals with more adaptive styles and well developed social skills may form better alliances with their therapists and have better prognoses according to Del Re et al. [76]. In such instances, it is unclear whether the alliance-outcome relationship is influenced more by the patient’s characteristics or something offered by the therapist. Furthermore, the differences in therapist skills and competencies between psychotherapy and physical rehabilitation professionals may affect how TA works in practice. For example, the application of electrophysical agents, manual therapy, exercise and physical activity is commonly associated with therapeutic procedures in PT and OT practice. Fuentes et al. [21] focused on empathetic communication and reported that the effect of TA on pain modulation in patients with chronic low back pain was enhanced when applied with active interferential current and their interaction may produce clinical benefits.

There was also a dearth of information on how the themes identified could be developed as soft skills that are practical for therapists to learn and adapt in clinical practice. Murray et al. [77] showed that physiotherapist training using self-determination theory based communication skills training improved perception of autonomous support among patients with low back pain. Similarly, the study by Fuentes et al. [21] highlighted how physiotherapist communication skills training based on empathy and roles and responsibilities can be used to enhance the patient-therapist relationship. In OT literature, the Intentional Relationship Model [78] was developed to increase occupational therapist’s capacity for developing skills in therapeutic use of self or TA using self-reflection guide on therapeutic modes. Taylor et al. [79] examined occupational therapists use of self according to the IRM when interacting with anxious or depressed patients.

Several of the TA measures identified are yet to be validated in patients with MSK conditions and some require further development before adaptation to MSK practice. Due to the complex nature of TA, available outcome measures were based on diverse TA themes. Only one measure covered all the themes of TA reported in this scoping review; the PRES [41]. The WAI [39, 40] was the most reported measure of TA and developed using Bordin’s model [8]. However, Hall et al. [80] showed that measures developed from psychotherapy such as the WAI [39, 40] exhibit a ceiling effect and require re-contextualization for suitable use in MSK practice. Several measures identified also had no evidence of psychometric evaluation which further limits applicability in MSK practice. Furthermore, some of the studies reviewed used outcome measures based on the construct of satisfaction [29, 81–83] to evaluate TA. It is unclear if these measures were assessing TA or satisfaction or both. A combination of measures may provide a more exact assessment of TA.

Our synthesis of the evidence on the impact of TA on adherence in MSK practice also focused on the relationship between TA and exercise adherence based on broad findings showing correlation between TA and adherence in several disciplines including medicine [84], psychotherapy [85] and physical rehabilitation [61]. However, the findings from the systematic review on adherence by Hall et al. [61] only reported a correlation between TA and cardiac and/or neurological rehabilitation. This scoping review showed that TA exerts diverse influence on treatment adherence as its predictor, moderator and mediator mostly in PT studies. Further studies are required to appraise the evidence in OT discipline. It is pertinent to elucidate moderators and mediators of RCTs because studying experimental intervention effects is unable to explain the mechanisms of intervention success or identify participants who benefit most from an intervention [86]. Such studies provide a key step to guiding interpretation of trials and design of future interventions. TA was also correlated with improved pain, reduced disability, and higher satisfaction in MSK PT practice [21]. TA was found to be more strongly associated with disability and function compared with pain outcomes in chronic LBP [87]. In addition, an identifiable “practitioner effect” was documented in MSK pain intervention trials [87]. Clearly, the context in which PT interventions are offered has the potential to dramatically improve therapeutic effects [21]. Unfortunately, the adherence literature is plagued by lack of robust outcome measures [88, 89] and calls to question the impact of TA on adherence in MSK practice.

### Study limitations

This scoping review utilised rigorous and transparent methods throughout the entire process. To ensure a broad search of the literature, the search strategy included nine electronic bibliographic databases, the reference list of thirty five different articles and ten relevant organizations. The relevant screening and data characterization forms were screened by two reviewers as needed prior to implementation. The greatest challenge in conducting a review in a broad and complex field like therapeutic alliance is not data collection but summarizing the data. Current views on scoping methodology advocate engaging a large inter-professional team at every stage of process to improve the quality of the decision making [35]. Unfortunately, due to time and financial constraints the authors were not able to build such a team for this review. Nonetheless, the authors were careful to use an iterative approach to clarify concepts and revising questions and themes with increased familiarity with the literature. Due to the language limit, we could have excluded studies that have direct relevance to the purpose of this review.

### Research opportunities and recommendations

Future research needs to focus on a clear conceptualization of TA in MSK rehabilitation with clear definition of terms in view of the broad complexity of TA. This approach has been proposed for other complex aspects of health such as quality of care [90]. Similarly, TA measures used in MSK PT and OT practice and the construct they assess need to be well-defined with evidence of psychometric properties. Furthermore, studies are required to increase therapist capacity at developing soft skills for enhancing TA in clinical practice. If these issues remain unaddressed, patients may continue to struggle to meet their rehabilitation potentials [63].

## Conclusions

This scoping review maps out the available literature on TA conceptualization, measures and insights into professionals’ and patients’ experiences and perceptions of TA in MSK rehabilitation. It appears that enhanced TA has some beneficial effects on treatment adherence. The limitations identified in existing literature provides a roadmap for designing future studies aimed at addressing key gaps identified in the TA literature. We propose further research focused on developing a physical rehabilitation themed framework of TA, psychometric testing of existing TA measures and designing trials to investigate the effect of therapist TA training on long term treatment outcomes and treatment adherence in MSK practice.

## Additional files

Additional file 1: Characteristics of studies included in the scoping review. Study information from each article included in the review (DOCX 133 kb)

Additional file 2: Therapeutic Alliance Terms. Description of therapeutic alliance terms detailed in included studies. (DOCX 63 kb)

Additional file 3: Perspectives on therapeutic alliance from participants. This file highlights the perspective and experiences of participants on therapeutic alliance. (DOCX 400 kb)
