# Supplementary material for: Characteristics of therapeutic alliance in musculoskeletal physiotherapy and occupational therapy practice: a scoping review of the literature
Source: BMC Health Serv Res. 2017 May 30;17:375. doi: 10.1186/s12913-017-2311-3 (PMC5450083; doi:10.1186/s12913-017-2311-3)
Supplement: Supplementary file 1 — Characteristics of studies included in the scoping review. Study information from each article included in the review (DOCX 133 kb) [file 12913_2017_2311_MOESM1_ESM.docx]

**Additional file 1: Characteristics of studies included in the scoping review**

| **Author** | **Year** | **Country** | **Setting** | **Discipline** | **Aims** | **Design** | | **Participants**  **(Patients or**  **Therapists)** | **Codes (Themes)**  **(subcategories)** | **Findings** |
| --- | --- | --- | --- | --- | --- | --- | --- | --- | --- | --- |
| **QUANTITATIVE STUDIES** | | | | | | | | | | |
| ***Adamson et al, (1s)*** | 1994 | Australia | Primary care  Private practice | OT | To explore the attitudes, values and orientation to professional practice of occupational therapists and the extent to which attitudes towards professional practice were related to demographic variables (age, gender, years of professional practice, level of seniority and workplace setting, whether public or private) was examined. | Correlational  (Survey) | | OTs  (n=378) | Knowledge  Techniques  Client characteristics  Client responsibility  Holistic  Attitudes  Approach to professional practice | Attitudes reflecting the new public health model emphasising client-OT interaction, client responsibility, and holistic attitudes toward healthcare prevailed among the studied OTs. Older (>30 years) and more experienced OTs (>6 years) endorsed a humanistic approach to therapy to a greater extent and greatly emphasised promoting the resources of the clients. |
| ***Ambady et al, (2s)*** | 2002 | USA | Primary care | PT | To explore the link between health care providers’ patterns pf nonverbal communication and therapeutic efficacy. | Descriptive study | | Geriatrics  (n=48)  PT (n=11) | *Study 1*  Communication; verbal and nonverbal | *Study 1*  Distancing behaviour was associated with short and long term decrease in ADL and short term increase in confusion and depression. Positive affect predicted short-term decrease in confusion and nervous and professionalism long term decrease in mobility.  *Study 2*  Facial expressiveness (smiling, nodding and frowning) communicates empathy and concern for clients and thus promotes patient satisfaction and health improvement. |
| ***Azoulay et al, (3s)*** | 2005 | Canada | Outpatients  Primary care | PT | To determine (1) patient – physical therapist and patient – physician agreement on clinical management of LBP,  (2) patient perception of agreement between physical therapist and physician, (3) association between agreement and outcome (return to work, self-perceived disability). | Pilot Randomized controlled trial | | Acute and chronic low back pain (n=35) | Perceived agreement | Nearly all patients (97.1%) agreed with the physical therapist and all believed the physical therapist was providing the treatment the physician would have thought appropriate. Disagreement was not associated with greater time off-work or greater self-perceived disability. |
| ***Baker et al, (4s)*** | 2001 | USA | Acute care | PT | To examine whether therapists seek to involve patients in goal setting and how this achieved. | Correlational | | Orthopaedic condition  (n=52)  PT (n=22) | Congruence  Communication  Relational aspects  Influencing factors | The therapists stated that they believed that it is important to include patients in goal-setting activities and that outcomes will be improved if patients participate. |
| ***Bassett and Petrie (5s)*** | 1999 | New Zealand | Private practice | PT | To test the effect of treatment goals on patient compliance with physiotherapy exercise programmes. | Randomized controlled trial | | Unspecified upper and lower limb injury  (n=74)  PT (n=17) | Agreement on goals | Collaboratively set goals appear to lead to a higher level of treatment compliance than physiotherapist-mandated goals |
| ***Beattie et al (6s)*** | 2005 | USA | Private practice | PT | To determine the validity of measures obtained from the MedRisk Instrument for Measuring Patient Satisfaction with Physical Therapy Care (MRPS) to differentiate between patient satisfaction with internal and external factors. | Cross-sectional Survey and  Measurement study | | MSK conditions (n=1449); Lower extremity (472),  Lumbar/ Thoracic spine (406),  Upper extremity (243),  Cervical spine (222), Hand/wrist (10) | Respect  Communication (listening, clear explanation) | Confirmatory factor analysis supported a good to excellent model fit for the internal and external factors. The SEM for the 2 factors was 0.19 and 0.24, indicating a low degree of measurement error. Both factors had high significant correlation with global measures of satisfaction. |
| ***Besley et al (7s)*** | 2010 | New Zealand | Private practice | PT | To explore client and PT perspectives of the acceptability and usability of the Working Alliance Inventory-Short form and Helping Alliance Questionnaire-Version 2, assess the test-retest reliability and explore their convergent and face validity. | Correlational (Survey) | | General  (n=22)  PTs (n=8) | Goal  Bond  Tasks | WAI and HAQ-II was acceptable and usable, and had good convergent validity (correlation coefficient of 0.72 (p<0.001) and 0.87 (p<0.001) for client and therapist). Some items were irrelevant, inappropriate, or missing; indicating the measures and may lack some conceptual clarity. |
| ***Bliss (8s)*** | 2010 | USA | Outpatient | PT | To examine psychosocial variables like attachment style, depression and the working alliance as predictors of treatment outcomes in the context of chronic pain patients receiving PT. | Descriptive study | | Chronic knee pain  (n=59) | Goals  Tasks  Bond | WAI scores were significantly positively correlated to compliance  (24% of the variance in compliance). |
| ***Burns et al (9s)*** | 1999 | USA | Occupational health | PT/OT | To investigate associations among patient hostility, anger expression, depressed mood, and quality of working alliance between patient and PT or OT in a work hardening program and examine whether patient characteristics predicted both patient and therapist accounts of working alliance quality. | Descriptive (Comparative, longitudinal) | | MSK pain from workplace injuries  (n=71)  PTs, OTs (n=7) | Working alliance  Patient hostility  Anger expression  Depressed mood | Age, pain duration, and number of pain-related surgeries were not related significantly to WAI-Patient or WAI-Therapist scores Therapist reported their poorest alliances with patients who were both depressed and expressed anger. Patient and therapist evaluations of the alliance were only marginally related. |
| ***Chan et al (10s)*** | 2009 | Hong Kong | Outpatient | PT | To investigate the impact of physiotherapists’ autonomy-supportive behaviours on patients’ motivation and rehabilitation adherence | Correlational (Retrospective cohort) | | Anterior cruciate ligament injury  (n=115) | Needs-supportive  behaviour | Adherence was predicted positively by autonomous treatment motivation. Autonomous treatment motivation was associated positively with autonomy support from PT. Autonomous treatment motivation fully mediated the effect of PTs’ autonomy support behaviours on adherence. |
| ***Chan and Can (11s)*** | 2010 | Turkey | Primary care  Outpatient | PT | To evaluate patients’ adherence to home exercise programs in PT practice and to learn which factors of patients’, therapists’, care or surroundings influence on patients’ adherence to home exercises. | Correlational (Survey) | | Orthopaedic Sports injury, Hand therapy and Rheumatology  (n=82) | Communication  Relationship  Monitoring exercise | Motivation (p<0.035), role of exercise (p<0.044), understanding (p<0.035), reassessment (p<0.005) verbal explanation (p<0.024), visual explanation (p<0.035), overall explanation qualities (p<0.002) and satisfaction with PT (p<0.004) had a strong effect on adherence. |
| ***Cheing et al (12s)*** | 2010 | USA | Outpatients | PT | To report the preliminary  validation results for the Pain Rehabilitation Expectations  Scale (PRES). | Descriptive (Measurement) | | Chronic Low back pain (n=50) | Expectation | The 3 subscales of the PRES (working alliance, proxy efficacy, and motivation/  expectation) were internally consistent, with Cronbach’s  a reliability coefficients ranging from 0.93 to 0.96. Proxy efficacy was found to be related to working alliance, and working alliance was positively related to client motivation and expectations. |
| ***Chen et al (13s)*** | 1999 | USA | Rehabilitation | PT/OT | To investigate factors from three models; MOHO, HBM, HLC for predicting increased compliance and satisfaction with home exercise programs. | Correlational (Prospective cohort) | | Upper-extremity orthopedic impairment (n=62) | Satisfaction | Only perceived self-efficacy and IHLC significantly contributed to compliance behavior (p<0.01). Therapists need to provide clear verbal and written instructions and review prescriptions with patients to identify and correct misunderstanding. |
| ***Cole and McLean (14s)*** | 2003 | USA | Various | OT | To determine practicing OTs’ perceptions about how the therapeutic relationship is currently defined and used in today’s health-care environment. | Descriptive (Survey study) | | OTs  (n=129) | Therapeutic alliance | TA is related to functional outcome. Definitions of TA included words and phrases expressed in the literature and by experts. There are differences in perceptions of TA among pediatric, adult, and geriatric specialty subgroups. Most OTs learned skills in developing TA during clinical practice rather than during professional education. |
| ***Crooks et al (15s)*** | 1998 | UK | Primary care | PT | To report the problem experienced with patient engagement in physiotherapist-led groups undertaking either an aerobic exercise or a stretching and relaxation program. | Quasi randomized controlled trial | | MSK disorders (n=228)  LBP (52),  Neck pain (30),  Lower limb pain (25), Shoulder pain (12) | Support  Advice | Listening to problems not directly related to physical improved adherence.  Encouragement, suggesting alternatives and active involvement, individual feedback, use of visual aids increased engagement. |
| ***do Nascimento et al (16s)*** | 2014 | Brazil | NS | PT | To investigate the impact of the therapeutic alliance in the recruitment of the deep abdominal muscles | Descriptive (Observational cohort) | | Nonspecific low back pain (n=12) | Bond  Agreement on goals  Agreement on tasks | The therapeutic alliance has no association with muscle recruitment in the short term. However, although there were no changes in muscle recruitment after the intervention program, the level of pain and disability was reduced. |
| ***Eklund et al (17s)*** | 2015 | Sweden | Primary care | OT | To investigate the working relationships, as perceived by clients and therapists in the Redesigning Daily Occupations (ReDO^TM^) program, and its relationships to return to work and satisfaction with the relationship | Quasi randomized controlled trial | | Neck pain  (n=37)  OTs (n=37) | Working relationship | REDO clients gave higher rating of TA due to collaboration and client-centred care. Clients rating of TA was not associated with RTW. No relationship between therapists’ rating of TA and satisfaction.  OT rating showed a statistically significant association with RTW at 12-month follow-up, but not at 6-mth follow-up or completion of intervention. |
| ***Ferreira et al (18s)*** | 2013 | Australia | Primary care | PT | To investigate whether the therapeutic alliance between physical therapists and patients with chronic low back pain predicts clinical outcomes. | Descriptive  (Retrospective observational) | | Chronic low back pain  (n=182) | Goal  Task  Bond | The therapeutic alliance was consistently a predictor of outcome for all the measures of treatment outcome. |
| ***Foster et al (19s)*** | 2010 | UK | Primary care | PT | To investigate the relationship between patient and therapist preferences and expectations and clinical  outcomes in a trial of exercise and acupuncture | Randomized controlled trial | | Knee osteoarthritis  (n=325) | Expectation | There was no evidence of a relationship between patients’ treatment preferences or expectations and pain reduction. We found weak evidence, from secondary outcomes, that patients’ expectations, both general and treatment-specific, are related to clinical outcome from exercise and acupuncture. |
| ***Fuentes et al (20s)*** | 2014 | Canada | Primary care | PT | To compare the effect of enhanced versus limited therapeutic alliance on pain intensity and muscle pain sensitivity in receiving either active or sham interferential current therapy | Randomized controlled trial | | Chronic low back pain (n=117) | Empathy  Caring  Encouragement  Warmth  Support | There was very little association between TA and differences in expectancies (4% of variance in TA explained by expectations) |
| ***Gleeson et al (21s)*** | 1991 | Australia | Outpatient | PT/OT | To develop policies and procedures about management of patient non-attendance in OT | Descriptive (survey study) | | Various including hand injuries and rheumatology in outpatients (n=100)  PTs (n=6) | Nonadherence | Some therapists saw non-compliance as the result of a need to develop personal skills (empathy, warmth, concern), demonstrating a feeling of responsibility for non-attendance. |
| ***Grannis (22s)*** | 1981 | USA | Community | PT | To examine the ideal physical therapist as perceived by the elderly patient and compared to the ideal held by the practitioner | Descriptive (Measurement study) | | Geriatrics  (n=32) | Humour  Care  Concern | Sense of humor, clear instructions, evaluation and care and concern were rated highly |
| ***Hall et al (23s)*** | 2012 | Australia | Primary care | PT | To investigate the psychometric properties of the working alliance inventory theory of change inventory (WATOCI) in physical rehabilitation. | Descriptive (Measurement study) | | Low back pain  (n= 206) | Goal  Task  Bond | Good internal reliability of the 9-item WATOCI. |
| ***Hills and Kitchen (24s)*** | 2007 | UK | Primary Care | PT | To examine the level of satisfaction that patients with acute and chronic musculoskeletal conditions have with their PT outpatient treatment | Descriptive (Survey study) | | MSK conditions – Fracture/Trauma and (n=135) Degenerative spinal or joint disease (n=144) | Expectation  Communication,  Therapist Organisation Outcome  Satisfaction | Correlation between the 6 subscales all achieved statistical significance. The highest correlation was between Communication and PT (0.711) and between Communication and Organisation (0.698) and Expectations and Outcome showed the lowest correlation. |
| ***Jackson et al (25s)*** | 2012 | Australia | One-on-one outpatient clinic-based rehabilitation | PT | To explore potential relationship s between clients’ “tripartite” efficacy constructs, their perceptions of relationship quality with their therapist, and their engagement in their exercise program (Study 1), and model actor and partner effects or clients’ and therapists’ efficacy beliefs in relation to perceptions of relationship quality (Study 2). | Descriptive study | | Lower limb degenerative MSK conditions: osteoarthritis, osteoporosis, bursitis  (n=68) | Relationship quality | *Study 1*  Favorable self-efficacy, other-efficacy, and RISE appraisals were each significantly related to relationship quality. The tripartite efficacy constructs were able to explain 60% of the variance in relationship quality. Increases in relationship quality were directly related to improved engagement, accounting for 18% of the variance in engagement ratings.  *Study 2*  Clients and PTs reported more adaptive relationship perceptions when they held strong tripartite efficacy beliefs (actor effects), and clients viewed their relationship in a more positive light when their PT was highly confident in their ability (partner effect). |
| ***Jensen and Lorish (26s)*** | 1994 | USA | Outpatient | PT | To integrate concepts from re- search, theory, and practice are integrated into a Process Model for Patient-Practitioner Collaboration for use in clinical practice | Descriptive (Survey study) | | Rheumatoid arthritis,  Osteoarthritis  Low back pain  (n=305)  PT (n=568) | Caring  Respect  Concern  Attention | Pleasing the PT was a reason for adherence to exercises prescribed |
| ***Kersten et al (27s)*** | 2012 | New Zealand | Primary care | PT/OT | To examine the internal construct (factorial) validity of the CARE in the assessment of the patient-therapist relationship | Descriptive (Measurement study) | | Hip and knee replacement  (n=213)  41%-hip 59%-knee | Therapist empathy | The CARE measure satisfies strict standards for internal construct validity, demonstrated by a fit to the Rasch model, so allowing for an interval scale transformation when required. |
| ***Kerssens et al (28s)*** | 1999 | Netherland | Private practice | PT | To study the effectiveness of a training program for the enhancement of patient education skills in PT. | Descriptive study | | Low back pain  (n=130)  PT (n=19) | Instruction  Problem solving  Skill & Competence | Training based on strategies suggested by Sluijs et al was not effective in teaching PTs to have a more individual approach deemed important to improve adherence and self-efficacy |
| ***Knight et al, (29s)*** | 2012 | Australia | Private practice | PT | To use a questionnaire based on the discrepancy model to assess the factors contributing to satisfaction and dissatisfaction with private outpatient PT services;  To test the applicability of the ‘‘consumer model’’ to PT practice, and to identify the criteria used to assess quality. | Descriptive  (Survey study) | | MSK disorders (n=292)  Lower back; 17.3%, Knee; 12.5%, Neck; 9.3%, Shoulder; 9.3%, Ankle; 8%, Legs; 6.1%  Hand; 1.6%  Elbow; 1.3%  Hip; 1%,  Multiple areas: 23.4% | Empathy Friendliness  Clear explanation  Understanding  Knowledge exchange  Attention | Poor professional behavior, discourtesy and uncaring attitude dampens perception of PT service quality. Courtesy, friendliness and respect by PT improves perception of PT service quality. |
| ***Levy et al (30s)*** | 2008 | UK | Private practice | PT | To investigate the relationship between perceived autonomy support, age, and rehabilitation adherence among sports-related injuries. | Correlational (Prospective cohort study) | | Tendon-related injuries (n=70)  Ankle (41%),  Knee (28%), Shoulder (20%),  Elbow (11%) | Needs-supportive behaviour | High autonomy support from PT was related to better clinic-based adherence and attendance particularly among older patients but not to home-based adherence. Age was related to all adherence indices and moderated the relationship between perceived autonomy support and clinic-based adherence. |
| ***Lysack et al (31s)*** | 2005 | USA | Inpatient rehab | PT/OT | To compare computer-assisted video instruction and routine rehabilitation practice on compliance and satisfaction with home exercise. | Randomized controlled trial | | Total hip and knee replacement  (n=40)  21 – hip  19 – knee | Exercise performance  Patient-therapist relationship  Satisfaction | There was no statistically significant benefit to be realized in either patients’ self-reported compliance, relationship to therapist or satisfaction with the use of the video exercise instruction. |
| ***Medina-Mirapeix et al (32s)*** | 2015 | Spain | Outpatient | PT | To describe the development and initial psychometric evaluation of a ﬁxed-length questionnaire about the experiences of patients receiving physical therapist treatment in post-acute outpatient settings | Descriptive (Measurement study) | | Chronic pain (MSK)  (n=465)  Lower back injury; (7.2%)  Upper limb fracture; (30%)  Lower limb fracture; (39.3%)  Shoulder injury; (12.5%)  Knee injury; (11%) | Emotional support  Sensitivity  Providing information and education  Interruptions during care delivery  Waiting time for treatment  Safety | 7-factor structure with 3 factors viewed as professionals’ attitudes and behavior (providing information and education, sensitivity to patients’ changes, and emotional support) and 4 factors reﬂecting organizational environment (duration of attendance, interruptions during care delivery, waiting times, and patient safety). Item-scale correlations; (0.70 to 0.93). Cronbach α (0.70 to0 .87). ICC; (0.57 to 0.80) |
| ***Murray et al (33s)*** | 2015 | Ireland | Primary care | PT | To examine the effects of communication skills training on physiotherapists’ supportive behaviour during clinical practice | Randomized controlled trial | | Chronic low back pain (n=24)  PTs (n=24) | Communication (Need-supportive) | There was a large between-arm difference in needs-support scores with intervention arm PTs rated as significantly more supportive than control arm PTs |
| ***Nierdeman et al, (34s)*** | 2011 | Switzerland | Primary Care | OT | To evaluate whether individualised, resource oriented joint protection education; Pictorial Representation of Illness and Self Measure (PRISM-JP) in patients facilitates joint protection acquisition and adherence more successfully, compared to conventional joint protection education (C-JP). | Randomized controlled trial | | Rheumatoid arthritis  (n=53) | Perceived relationship | PRISM improved patient-clinician communication and is feasible for occupational therapy. |
| ***Osmotherly and Higginbotham (35s)*** | 2004 | Australia | Outpatient | PT | To understand how the Triandis model of behaviour intention, a component of the theory of social behaviour, to develop and validate a questionnaire measuring a patients’ intentions to perform a prescribed home exercise program, and explore which variables of the model are significant predictors of patient s’ intentions. | Descriptive (Cross-sectional study) | | Rotator cuff pathology, low back (n=234) | Intention to exercise | Questionnaire demonstrated good internal consistency and reliability and the ability to predict intention to exercise. It explained 21.77% of the variance to exercise. |
| ***Roberts and Bucksey (36s)*** | 2007 | UK | Primary care (acute) | PT | To measure the content and prevalence of the verbal and nonverbal communications that occur between physical therapists and patients in an outpatient setting | Descriptive (Prospective, observational study) | | Chronic back pain  (n=21)  PT (n=7) | Communication | Content behaviors (taking history, giving advice) comprised 52% of verbal communications. Touch by PT (54%) and eye gaze by patients (84%) were the most prevalent nonverbal behaviors. |
| ***Sluijs et al (37s)*** | 1991 | Netherland | Private practice | PT | To describe differences between physical therapists in terms of the attention they pay to educating patients. | Descriptive study | | Various: frozen shoulder, tennis elbow, knee arthritis, lumbago, sprained ankle, osteoporosis (n=25) | Reinforcing patients’ performance  Showing concern  Showing interest | PT belief in patient’s level of compliance and amount of time spent with the patient improved the TA relationship. |
| ***Sluijs et al (38s)*** | 1993 | Netherland | Private practice | PT | To investigate whether patent compliance was related to characteristics of the patient of the patient’s illness, attitude or physical therapist’s behaviour. | Descriptive (Cross-sectional study) | | Radiating back pain and non-radiating back pain; (27%)  Neck and shoulder pain; (17%)  Trauma and postoperative; (13%)  Multiple; (5%)  (n=84)  PT (n=300) | Interest  Active Involvement  Pain concern  Patient participation  Reinforcing performance  Instructions | Barriers perceived and encountered, lack of positive feedback, and degree of helplessness were the 3 main factors. Noncompliance is more strongly related to the characteristics of the illness than to the illness, a bad prognosis, and much complaint is positively related to compliance. |
| ***Taylor et al (39s)*** | 2011 | USA | Various  34% - inpatient  22.6% - outpatient rehabilitation  23% - school settings  9.9% - home health  9.6% - private practice  0.9% - other | OT | To explore the therapist’s use of self by examining the use of different modes of interacting with clients and whether modes vary when the characteristics of clients vary | Correlational (Prospective survey design) | | OTs  (n=563) | Interpersonal modes  Instructing, (I)  Empathizing (Em)  Collaborating, (C)  Problem-solving, (P)  Encouraging (En) | Modes used often from most to least were: Em, C, P, I and Em and did not differ according to client population. OTs who experienced more difficult behaviours and emotions reported higher levels of using all modes but more likely to report using I and P modes. P, C, and E were used more for depressed clients were. P was used most for anxiety. |
| ***Thomson et al (40s)*** | 1997 | UK | Outpatient | PT | To measure the empathy in a clinical and nonclinical settings with the purpose of investigating any association between years of clinical practice and levels of empathetic understanding | Correlational (Measurement study) | | General  (n=8)  PT (n=8) | Empathy | Empathy is not directly correlated with PT level of experience |
| ***Tousignant et al (41s)*** | 2011 | Canada | Primary care (acute) | PT | To measure the satisfaction of both patients and healthcare professionals with the technologies and services provided during in-home telerehabilitation as an alternative to conventional rehabilitation after discharge from total knee arthroplasty surgery. | Descriptive (Survey study within a randomized controlled trial) | | Total knee arthroplasty  (n=48) | Satisfaction | Patients perceived a good relationship with the PT despite in direct contact through telemedicine |
| ***Vong et al (42s)*** | 2011 | Hong Kong | Outpatient | PT | To examine whether the addition of motivational enhancement therapy (MET) to conventional PT produces better outcomes than PT alone | Randomized controlled trial | | Chronic low back pain (n=88) | Bong  Goals  Task | Adding MET (Motivational Enhancement Therapy) to conventional PT produced significantly better compliance, and improved pain, disability, motivation, lifting capacity and general health. |
| ***Wright et al (43s)*** | 2014 | Canada | Outpatient | PT | To identify which factors best explain non-adherence to home rehabilitation for patients with MSK injuries. | Descriptive (Cross sectional study) | | Musculoskeletal injuries  (n=87) | Trust  Communication  Productivity  Consultation | Patients are most likely to adhere to HRE when they perceive a positive relationship with their PT. |
| **QUALITATIVE STUDIES** | | | | | | | | | | |
| ***Aguilar et al (44s)*** | 2012 | Australia | Various  -Community  -University  -Private practice  -Rehabilitation  -Primary care | PT | To make a  preliminary identification of the values of the profession, by exploring the shared professional values | Ethnography  Naturalistic approach,  Constructivist paradigm | | PTs  (n=14) | The patient and patient-therapist  Partnership  -understanding and treating the individual  -patient-centred  -Trust  -Collaboration  Physiotherapy knowledge, skills and practice  -Managing limitations  -Mastery of skills and knowledge  -Team player  -Communicator  -Safe practice  -Getting the job done  -Evidence based practice  -Educator  -Professional  -Leadership  Altruistic values  -Honesty  -Respect  -Gong the extra mile  -Empathy  -Compassion  -Fairness  -Caring | The values that emerged went beyond philanthropic values, to values that guided every day practice, professional relationships and the responsibilities of being a professional. |
| ***Bamford and Walker (45s)*** | 2010 | UK | Primary care | OT | To explore the experiences of working age people with dominant side hand injury in terms of impact on their lives from the resulting loss of hand function, and the role that hand therapy plays in the rehabilitation process | Phenomenology  Semi structured interviews  Thematic analysis | | Distal radius and/or ulna fracture  (n=6) | Patient experience | OT input increased motivation and particularly for those who attended rehabilitation sessions in the department, the therapy was valued. |
| ***Bassett and Tango (46s)*** | 2002 | New Zealand | Private practice | PT | To understand and interpret Maori’s people’s experiences of being PT patients. | Phenomenology  Individual face-to-face semi-structured interviews | | Chronic MSK pain  (n=6) | Treatment experience | Positive interaction and good rapport with PT fosters empowerment and taking responsibility to adhere to the home exercise. Qualities of respect, cultural sensitivity, sense of comfort, openness, honesty, clear communication, use of visual aids, were appreciated by patients to contribute to exercise adherence. |
| ***Bunzli et al (47s)*** | 2016 | Australia | NS | PT | To investigate participants’ experience of cognitive functional therapy (CFT) by comparing participants who reported differing levels of improvement after participation in CFT, potentially yielding insight into the implementation of this approach. | Interpretive description  Cross-sectional non-interventional with interpretive description framework | | Chronic low back pain  (n=14) | Trust in therapist  Communication | Therapeutic alliance appeared to play a role in challenging  pre-existing beliefs. The establishment of a trusting relationship with the therapist appeared to be important in  facilitating effective communication in which individuals felt comfortable airing their concerns and doubts, with the underlying faith that the therapist had  their best interests at heart: |
| ***Campbell et al (48s)*** | 2001 | UK | Community | PT | To understand reasons for compliance and non-compliance with a home-based exercise regimen by patients with osteoarthritis of the knee. | Grounded Theory  Audio-taped interviews with content analysis  nested within a randomized controlled trial examining effectiveness | | Knee osteoarthritis  (n=20) | Barriers  Facilitators | Continued compliance depends on a person’s perception of their symptoms, the effectiveness of the intervention, their ability to incorporate it into everyday life and support from physiotherapists. |
| ***Cipriani et al, (49s)*** | 1999 | USA | Long term care | OT | To determine whether and how collaboration occurred in the therapeutic relationship, from the perspective of older adult clients receiving OT services. | Phenomenology approach  Semi structured interviews | | Various (n=6)  involving shoulder injury (n=1) and total hip arthroplasty (n=1) | Collaboration | Therapists need to be aware of the signals their clients send in order to establish an effective collaborative process. One perception was that collaboration doesn’t mean ‘‘equality’’ in all client-therapist interactions, but rather an information exchange and mutual respect between the client and the OT. |
| ***Cooper et al (50s)*** | 2008 | UK | Primary care | PT | To define patient-centredness from the patient’s perspective in the context of physiotherapy | Phenomenology  Semi-structured interviews with  Framework analysis | | Chronic low back pain (n=25) | Clear information  Listening  Influencing factors  Active involvement  Personalized therapy  Roles and responsibilities  Therapist competence and personality | Six key themes emerged as the dimensions that the participants perceived to be important for patient-centred physiotherapy:  Communication, individual care; decision-making; information; the physiotherapist; and organisation of care. Communication was the most important dimension, underpinning the five other dimensions as well as being a distinct dimension of patient-centred physiotherapy. |
| ***Coutu et al (51s)*** | 2013 | Canada | Rehabilitation | OT | To define and describe scenarios depicting the differences between clinical judgment, workers' representations about their disability and clinicians' interpretations of these representations. | Multiple Case-study using prospective semi-structured interviews with a clinician and worker dyad | | Workplace injuries (n=12)  Upper extremity (n=2)  Back (n=8)  Both upper extremity and back (n=2)  OT (n=5) | Clinician judgement  Worker representation | Clear problem identiﬁcation by clinicians was important to allow for the use of concrete and pragmatic strategies and congruence between the proposed strategy and workers representation were crucial. During rehabilitation, the objectives must be acceptable to both parties or the proposed strategy must, at least, make sense to the patient. |
| ***Crepeau and Garren (52s)*** | 2011 | USA | Private outpatient practice | OT | To illustrate important dimensions of the therapeutic relationship in a hand therapy patient-therapist dyad. | Case study of a therapist and patient dyad  Semi structured interviews | | Elbow dislocation  (n=1)  OT (n=1) | Therapeutic relationship | Combining technical knowledge and skills of hand therapy with OT principles of collaboration between therapists and patients, both patient and therapist can find meaning in the therapy experience. |
| ***Dahlgren et al (53s)*** | 2000 | Sweden | Private practice | PT | To describe and analyse PTs’ experiences of participation in Balint group training (BGT) as a means of learning and understanding the physiotherapist–patient relationship. | Interpretative phenomenology  Semi-structured interviews | | Chronic pain  PT (n=3) | Communication  Relationship | BGT and sharing the experiences of others may be considered a way of enhancing understanding of the patient encounter in clinical practice to the benefit of both PTs and patients. |
| ***Del Bano-Aledo et al (54s)*** | 2014 | USA | Outpatient | PT | To identify elements of physiotherapist-patient interaction considered by patients when they evaluate the quality of care in outpatient rehabilitation settings. | Grounded Theory  Focus group | | Upper and lower limb fractures,  Joint arthroplasty  Orthopaedic surgery  (n=57) | PT Service quality | Friendliness, respect, emotional supportive care, sensitivity, patient understanding, timely, regular and clear explanations, skill and competence are related to perception of service quality. |
| ***Ekerholt and Bergland (55s)*** | 2004 | Norway | Primary Care | PT | To elucidate patients’ experiences of the examination of the body given in Norwegian  Psychomotor Physiotherapy. | Grounded theory using open coding | | Psychomotor  and MSK disorder (n=10) | Establishing the relationship – empathy, trust, sufficient available time, calm, friendly, interest.  Professional skills – security and competence.  Personal boundaries –environment, respect. | The category “establishing the relationship” emerged from the subcategories getting to know the therapist as an empathic person and discovering professional skills.  Professional thoroughness and seriousness leads to established trust between patient and therapist.  Creating a sensitive and safe environment in vulnerable situations establish a foundation for a future therapeutic relationship. |
| ***Escolar-Reina et al (56s)*** | 2010 | Spain | Community | PT | To explore perceptions of people with chronic neck or low back pain about how characteristics of home exercise programs and care-provider style during clinical encounters may affect adherence to exercises. | Grounded theory  Focus groups | | Chronic neck or low back pain  (n=34) | Care-provider style  Characteristics of home exercise programs | Patient adherence to home-based exercise is more likely to happen when care providers’ style and the content of exercise programme are positively experienced. |
| ***Farin et al (57s)*** | 2011 | Germany | Primary Care | PT | To develop a patient-oriented and theory-based questionnaire on the communication preferences of chronically ill patients (KOPRA questionnaire) and to carry out psychometric testing of the instrument. | Grounded theory  Focus groups  Cognitive interviews | | Chronic low back pain (n=239) | Communication | In the physician version with a total of 32 items, there are four scales (‘‘Patient participation and  patient orientation’’, ‘‘Effective and open communication’’, ‘‘Emotionally supportive communication’’, and ‘‘Communication about personal circumstances’’) that are unidimensional, fulfill the demands for a 1-parameter IRT model, and are reliable (Cronbach’s alpha between .80 and .92). The psychometric properties with respect to nursing staff and therapists are slightly worse. |
| ***Gard et al (58s)*** | 2000 | Sweden | Primary care | PT | To investigate how many and what verbally expressed emotions physiotherapists state during interviews between PTs and patients. | Case study  Individual interviews  Cross-case analysis | | PTs (n=10) | Emotional intelligence (EI) | Interest was the most expressed emotion and stressed as the most important motivator in the development of skills and competencies and seen as a prerequisite for the development of EI. |
| ***Greenfield (59s)*** | 2006 | USA | Primary care | PT | To describe the experiences of five experienced PT to understand the nature of caring in their clinical practice. | Interpretative narrative  In-depth, open-ended interviews | | PTs (n=5) | Caring | Commitments to patient caring were reflected in receptivity to patients and willingness to displace own judgments and prejudices to understand patients’ motivations. |
| ***Greenfield et al (60s)*** | 2008 | USA | Primary care  Rehabilitation inpatient  Outpatient | PT | To determine patients’ perspectives of components of patient-centred PT and its essential elements | Phenomenology  Semi-structured interviews | | PTs (n=7) | Caring | Many participants viewed caring as a rules-based approach, core value and a moral orientation that guided their ﬁrst year of clinical practice |
| ***Gyllensten et al (61s)*** | 1999 | Sweden | Primary care | PT | To investigate expert PTs’ perception of important factors influencing the quality of the interaction in physiotherapeutic treatment. | Case study  Structured interviews  Cross-case analysis | | PTs (n=2) | Interaction | Interaction skills of the expert PT is essential for promoting a positive patient outcome and can be reinforced by reflecting on the patients’ experience. |
| ***Harrison and Williams (62s)*** | 2000 | UK | Outpatient | PT | To explore the power balance from both the patient’s and the PT’s perspectives. | Phenomenology  In-depth  semi-structured interviews  Content thematic analysis | | Unspecified MSK conditions (n=5)  PT (n=5) | Power balance | A complex interplay of patient, therapist and environmental variables was demonstrated.  Patients viewed themselves as having little or no control during the clinical encounter and their experiences did not reflect collaborative patient-centred |
| ***Harman et al (63s)*** | 2012 | Canada | Military | PT | To describe the approach used by a PT who led a rehabilitation programme for injured members of the military with chronic low back pain designed to enhance self-efﬁcacy and self-management skills. | Narrative study  In-depth audio- and video-recorded interviews.  Inductive analysis | | Chronic low back pain (n=12) | Rapport Trust  Support  Empathy | Using TA (rapport, trust, empathy) and behaviour change techniques, PTs can focus on the perceived importance of a behaviour change (need) and then shift to the patient’s self-efﬁcacy in the solutions phase. |
| ***Haywood et al (64s)*** | 2015 | UK | Primary care | PT/OT | To explore the CPD needs and issues for clinicians  working in MSK services; explore the perceptions of CPD held by people who  use MSK services; compare the above with current literature to discern any factors specific to MSK settings. | Interpretative phenomenology  Focus groups  Conference calls | | PTs (n=11)  OTs (n=2)  MSK condition (n=11; OA, RA, back pain) | Communication | Patients expected clinicians to listen to their stories and learn from them and demonstrate good communication skills with patients and other professionals. Some PTs believe that communication skills may not be a priority area for healthcare professionals. |
| ***Hinman et al (65s)*** | 2015 | Australia | Community | PT | To explore how stakeholders (patient, physiotherapists and telephone coaches) experienced, and made sense of, being involved in an integrated program of PT-supervised exercise and telephone coaching | Symbolic interactionism  Semi-structured individual interviews,  cross-sectional design | | Patients with knee pain  (n=6)  PT (n=10) | Active involvement | Patients appreciated personalised, genuine interest from PTs. A collaborative approach, with defined roles for PTs and communication strategies and sharing information was identified as important for effectiveness in the exercise program. |
| ***Hurley et al (66s)*** | 2010 | UK | Primary care  Rehabilitation | PT | To explore the health beliefs, experiences, treatment and expectations of people with chronic knee pain, and investigate if, how and why these changes after taking part on an integrated exercise-based rehabilitation programme; ESCAPE (Enabling Self-management and Coping with Arthritis Knee Pain through Exercise) | Phenomenology  Semi-structured interviews  Thematic analysis | | Chronic knee pain  (n=29) | Partnership | Interpersonal qualities (care, support, guidance) and professional skills of the PT were as important to the success of the program as the program content. |
| ***Karnad and McLean (67s)*** | 2011 | UK | Primary care | PT | To explore PT’s perception of exercise adherence and interventions used in clinical practice. | Interpretative Phenomenology  Semi structured interviews | | PTs (n=5) | Patient-therapist collaboration | Most PTs believe that clear communication, faith in the PT, realistic treatment plans, shared goals and pain education are important for adhering to exercise. |
| ***Kidd et al (68s)*** | 2011 | New Zealand | Outpatient | PT | To determine patients’ perspectives of components of patient-centred PT and its essential elements | Grounded theory  Semi-structured interviews | | MSK disorders and accident-related rehabilitation  (n=8) | Patient centred care | Patient centred care model locates the patient at the centre of the professional relationship, and supports the notion that an understanding of the patient’s perspective should underpin good practice in an equal therapeutic relationship. |
| ***Kumlin and Kroksmark (69s)*** | 1992 | Sweden | Inpatient  Outpatient | PT | To delineate PT’s conceptions of establishing therapeutic relationships with patients and conceptions of activating the resources of the patients. | Grounded Theory  Individual semi-structured  in-depth interviews | | PTs (n=10) | Therapeutic relationship | PT knowledge, understanding of establishing relationships with patients was described as personal, private and non-thematic through a relationship based on a dialogue aimed at discovering the patient’s conceptions of their problems and strategies to solve them instead of perceiving the PT as the authority. |
| ***Larsson et al (70s)*** | 2010 | Sweden | Various:  Rehabilitation  Primary care (orthopedics, rheumatology, surgery)  Occupational health  Acute care  Habilitation | PT | To describe how PTs experience client participation in physiotherapy  interventions | Phenomenology  Individual tape-corded interviews | | (PTs=11) | Decision making  Goal setting | Together the client and the PT |
| ***Liddle et al (71s)*** | 2007 | New Zealand | University Health Centre | PT | To explore the experiences, opinions and treatment expectations of patients to identify what components of treatment they consider as being of most value. | Narrative  Focus groups | | Chronic low back pain  (n=18) | Treatment expectation  Influencing factors | To enhance treatment effectiveness, participants welcomed the introduction of individually tailored advice and exercise programmes, with supervision and follow-up support, along with a  better understanding of the physical and emotional impact of chronic LBP by practitioners |
| ***Littlewood et al (72s)*** | 2014 | UK | Private practice | PT | To explore potential barriers with participants involved in a pilot self-management loaded exercise intervention | Phenomenology  Semi-structured interviews  Framework analysis | | Rotator cuff tendinopathy (n=6)  PT (n=2) | Self-management | Ongoing support (providing feedback, pro-active follow-up, and stimulating further engagement) with exercise programs and early and appreciable response to therapy were influential on successful outcomes. |
| ***Mann and Gooberman-Hill (73s)*** | 2011 | UK | Primary care | PT/OT | To explore the opinions of patients and health professionals about the provision of health care and possible service improvements. | Phenomenology  Focus groups | | Osteoarthritis  PTs (n=1)  (OTs (n=2) | Communication  Partnership  Influencing factors  Congruence  Roles and responsibilities | Improved information about disease would guide treatment expectation, clarify needs, and improve patient confidence for self-management. |
| ***May (74s)*** | 2001 | UK | Community | PT | To describe the aspects of physiotherapy care which back pain patients consider important. | Phenomenology  Semi-structured interviews  Framework analysis | | Back pain  (n=126) | Satisfaction | Patients appreciates a PT that is friendly, empathetic, respectful, listens actively, offers personalised care, want someone skilled and knowledgeable, able to provide a wide range of information, allows patient involvement in evaluation of treatment and promotes active coping with problem. |
| *Medina-Mirapeix et al (75s)* | 2003 | Spain | Outpatient Rehabilitation | PT | To identify the beliefs and perceptions of patients with chronic neck and low back pain that influence adherence to home exercise during exacerbation and/or remission of pain. | Modified Grounded Theory  Semi-structured interviews | | MSK conditions  (n=55) | External factors | Participants perceived the quality of the rehab service on the basis of experiences with environmental factors including 3 physical; facility design, ambient conditions, and social factors, and 4 organizational factors; duration of attendance, interruptions during delivery, waiting times and patient safety. |
| ***Norby and Anna-Lena (76s)*** | 1995 | Sweden | Various | OT | To get a picture of how OTs perceive the therapeutic encounter with their patients | Grounded Theory  Semi-structured interviews  Conceptual and analytic framework | | OTs  (n=16) | Therapeutic encounter | The egalitarian approach in the tentative model addresses a symmetric relationship, i.e., the patient is regarded as a rational collaborator with the therapist, free to choose or reject therapeutic services. The OT is a teacher and motivator in the therapy process. |
| *Øien et al*  *(77s)* | 2011 | Norway | Outpatient | PT | The purpose of this study was to describe communicative  patterns about change in demanding physiotherapy  treatment situations. | Multiple case study with cross-case analyses  (Longitudinal)  Semi-structured interviews  Focus groups with video-recording | | Chronic MSK pain (back and neck)  (n=12)  PTs  (n=6) | Communication | One main communicative pattern was identified: seeking for common ground – demanding negotiating  process. This pattern was interrupted by short episodes of two types of challenges; the pattern of ambivalence and uncertainty, and the pattern of impatience and disagreement. |
| ***Owen OG and Goodge P (78s)*** | 1981 | UK | Outpatient  General  Community | PT | To examine the validity of Bryne and Long’s communication model to analyse a sample of PT’s communication behavior. | Phenomenology  Content analysis | | PTs (n=11) | Communication | Two clusters of behaviors were obtained: 1 (direct questions, advising, directing, rejecting), 2 (empathizing, constructive feedback, counselling). The 2-dimensional model seems to resolve what have sometimes been conflicting approaches to interactions between health professionals and patients.  It seems that physiotherapists'  interactions are best described by one dimension of direction and advice giving and a second,  independent dimension of a counselling type of communication. |
| ***Palmadottir (79s)*** | 2006 | Iceland | Rehabilitation | OT | To explore the clients’ perceptions and experiences of the relationship that they formed with their OT in the context of rehabilitation. | Phenomenology  Unstructured interviews | | Multiple conditions (n=20) | Client-therapist relationship | Participants expressed a great deal of satisfaction with the relationship that they had formed with their primary OT. Of the 7 dimensions identified in this study, 5 were experienced as positive (concern, direction, fellowship, guidance, and coalition), 1 as negative, and 1 as neither positive nor negative. |
| ***Papi et al (80s)*** | 2016 | UK | Primary Care | PT | To investigate clinicians’ views of health-related wearable technologies in the context of supporting osteoarthritis long-term management. | Grounded Theory  Inductive thematic analysis  Semi structured in-depth interviews | | PTs (n=4) | Communication  (listening, feedback)  Caring | Therapists believe that wearable technologies monitoring the patients would enhance rather tan interfere with the clinician-patient relationship by reassuring patients that they are receiving high-quality treatment. |
| ***Peiris et al (81s)*** | 2012 | Australia | Inpatient  Rehabilitation | PT | To investigate how patients receiving inpatient rehab experience physiotherapy and whether their experience differ after receiving extra Saturday physiotherapy | Grounded Theory  In-depth interviews  Thematic analysis and  triangulation | | Various  Total joint arthroplasty (n=7)  Fracture (n=4)  Amputation (n=1)  PTs (n=9) | Empathy  Caring  Friendly  Influencing factors | Patients associated physiotherapy with personal attributes of the physiotherapists, and interaction with staff and other patients during physiotherapy. The patient-therapist interaction was more important to the patient than the amount or content of their physiotherapy, but Saturday therapy changed patients’ perceptions of weekends in rehabilitation. |
| ***Petursdottir et al (82s)*** | 2010 | Iceland | Outpatient | PT | To increase knowledge and understanding of the experience of exercising among individuals with osteoarthritis and to determine what they perceive as facilitators and barriers to exercising. | Phenomenology  Semi-structured interviews | | Osteoarthritis; (n=12)  Hip/knee; (n=10)  Vertebral column; (n=9)  Hands; (6)  Other joints; (3) | Professional care | Encouragement, understanding, clear communication, sense of positive connection with PT were important for adherence to exercise. |
| ***Potter et al (83s)*** | 2003 | Australia | Private practice | PT | To identify the qualities of a ‘good’ PT and to ascertain the characteristics of good and bad experiences in private practice PT from the patients’ perspective. | Nominal group technique  Audio-taped group meetings | | General  (n=26) | Qualities of a good PT  PT experience | Communication ability was highly regarded by all groups of patients and good PT experiences were most often attributed to effective communication while bad experiences most often related to dissatisfaction with the service and poor PT communication. |
| ***Potter et al (84s)*** | 2003 | Australia | Private practice | PT | To identify a typology of the difficult patient in private practice PT and to determine strategies physiotherapists use, and would like to improve, when dealing with such patients. | Nominal group technique  Inductive content analysis | | PTs (n=37) | PT and patient expectation  Patient attributes  Managing difficult patients | The two areas physiotherapists found most difficult to manage were behavioural problems of patients and patient expectations. PTs highlighted communication and behaviour modification strategies as the two areas they would like to improve in to assist their work with difficult patients. |
| ***Rosa and Hasselkus (85s)*** | 1996 | USA | Rehabilitation | OT | To examine in depth data specific to the patient therapist relationship | Narrative  Individual interviews | | Various:  Acute care Geriatrics  Burns  Hand therapy, Work hardening  OTs (n=83) | Connecting with patients; Helping  Collaboration | Helping involved taking responsibility for what happens to the patient, getting patients to acknowledge and value progress, providing comfort and support and empathy. Working together involves shared responsibility and work, collaboration, |
| ***Rosa and Hasselkus (86s)*** | 2005 | USA | Various  Rehabilitation  Outpatient  Home care  Private practice  Acute care | OT | To examine the nature and meaning of therapist-patient interactions from the perspective of the therapist. | Narrative | | OTs (n=14) | Working together (finding common ground) on goals and expectations | OTs may resist negotiating differences with patients over therapy goals and expectations and instead rely on compatibility as the basis of ﬁnding common ground. It is importance for OTs to be open to negotiating differences with patients over goals and expectations, aware of the ideologies that may inﬂuence their practices, and. adequately prepared to deal effectively with the challenging interpersonal aspects of practice. |
| ***Slade et al (87s)*** | 2009 | Australia | Community | PT | To understand the factors that participants in exercise programs for chronic low back pain perceive to be important for engagement and participation | Grounded Theory  Tape recorded interviews  Focus group | | Chronic low back pain (n=18) | Help  Empowerment  Communication  Knowledge exchange | Participants prefer PTs to be non-judgmental, empathetic, patiently listen and consider wants, needs, and circumstances in designing exercise and collaboration and shared decision in making care plan. |
| ***Stenner et al (88s)*** | 2016 | UK | Primary care | PT | To explore how shared decision making and patient partnership are addressed by physiotherapists in the process of exercise prescription for patients. | Interpretative phenomenology  Philosophical hermeneutic approach | | Nonspecific low back pain, PTs (n=8) | Partnership | The findings revealed how PT practice often resulted in unequal possibilities for patient participation which were in turn linked to the physiotherapists’ assumptions about the patients, clinical orientation, cognitive and decision making processes. Three themes emerged:  1. I want them to exercise: define options, checking patient understanding and ability to implement the plan.  2. Which exercise? – the tension between evidence and everyday practice: interpret the evidence, exercise needs to be fun, depends on what I find,  3. Compliance-oriented more than concordance based: pinpointing barriers, |
| ***Swardh et al (89s)*** | 2008 | Sweden | Primary care | PT | To explore and describe ways of understanding exercise maintenance among individuals with RA who had already started to exercise. | Phenomenology  Individual semi-structured interviews | | Rheumatoid arthritis  (n=18) | Support | The way patients perceive, discuss and experience the type of support (control, guidance, collaboration) and personal factors affected long-term exercise adherence. |
| ***Thomson (90s)*** | 2008 | UK | Primary care | PT | To explore the therapists’ perspectives on how therapist/patient interactions influence success or contribute to meeting the patient’s goals on a pain management program. | Ethnography  Semi-structured interviews | | Chronic MSK pain  (n=12)  PT (n=5) | Agreement on goals | Positive outcomes are related to interactions typified by assertiveness, negotiation, and critical reflection and poor outcomes due to unwilling and unrealistic interactions. PTs can create a collaborative framework when patients are willing to grasp the opportunity offered to them. |
| ***Thornquist (91s)*** | 1992 | Norway | Community | PT | To ascertain what happens in first encounters between patients and PTs | Narrative  Semi-structured interviews  Descriptive analysis | | Whiplash  Headache, dizziness, neck and shoulder myalgia  (n=2) | Communication | Two-way communication involves patient's views, comments and reactions during the whole examination and conclusion and actively seeking patient opinion on findings and evaluations communicate. This allows patients to received information on and contributed to the relevance of the findings, conclusion and choice of treatment |
| ***Veenhof et al (92s)*** | 2006 | Netherland | Primary care  Rehabilitation | PT | To understand why some patients who have received a behavioural graded activity program successfully integrate the activities into their daily lives and others do not. | Grounded theory  Open-ended, in-depth interviews | | Osteoarthritis  (n=12) | Agreement on goals | Active involvement of patients during intervention seems to relate to adherence but positive or former experience with PT had no effect on exercise adherence. |
| ***Wikman and Falthom (93s)*** | 2007 | Sweden | Primary care | PT | To describe patients’ experience of participation in and influence on rehabilitation with the focus on physiotherapy. | Grounded theory  In-depth  Interviews  Thematic analysis | | Various including orthopedics (n=6) | Active listening  Support  Encouragement  Active involvement  Trust  Influencing factors  Roles and responsibilities | The informants perceived that they were listened to and reinforced. Through information and support from the physiotherapist, the informants had learned to know themselves in body and mind. This was not, however, due to empowerment and options, but was the result of using a coping strategy that suited the traditional medical model. |
| ***Waters et al (94s)*** | 2016 | Australia | Outpatients | PT | To identify the factors influencing patient satisfaction with orthopaedic outpatient clinic services | Phenomenology  Crossectional  Focus groups  Interviews | | MSK conditions (n=10; knee OA, shoulder condition, foot pain, patella-femoral injury, ankle sprain | Empathy  Communication  Expectation  Trust  Relatedness  Time available | The focus group and interviews supported the notion of empathy  as an influential factor for patient satisfaction. Empathy appeared to reinforce interpersonal  aspects of the clinical interaction and was closely associated with caring behaviours. Patients described the importance of empathy within their experience of clinical assessment |
| ***Wilson et al (95s)*** | 2017 | UK | Primary care | PT | To investigate patients’ beliefs about, and experiences of, this type of treatment, and helpful and unhelpful experiences. | Interpretive phenomenology  Semi structured interviews | | Chronic pain MSK  (n=8) | Perceived relationship | The relationship between clinician and patient was a key factor in helping to understand and overcome emotional and physical difficulty. Openness and warmth of therapist transcends any conflict over approach and facilitated engagement in therapy. |
| **MIXED METHODS** | | | | | | | | | | |
| ***Favre et al (96s)*** | 2015 | Switzerland | Tertiary care | PT/OT | To understand patients’ and caregivers’ subjective perceptions and beliefs about care related pain (CRP) | Explanatory study | | Work, leisure or traffic accidents with chronic pain  (n = 20) | Communication  Empathy | Patients (46%) reported that richness of the means of PT: explanations and reassurance, collaborative dialogue and seeking feedback, encouraging and motivating patients, showing empathy and understanding help to alleviate CRP. Only 15% of caregivers invoked empathy |
| ***Freene et al (97s)*** | 2014 | Australia | Community | PT | To compare a PT-led home-based physical activity program to usual practice of community group exercise program to determine effectiveness in middle-aged adults for increasing physical activity levels over the short and long term. | Randomized controlled trial and focus groups with audio recorded interviews | | Sedentary adults with chronic disease including MSK  (n=28) | Participation enablers and barriers | Advice and support and individually tailored program from the PT and a good relationship with the instructor was important for continued participation in physical activity at home. |
| ***Kingston et al (98s)*** | 2014 | Australia | Primary care | OT | To determine if compliance and understanding of a home exercise program following a traumatic hand injury is improved when patients are provided with a DVD and a brochure when compared to using brochures only | Randomized controlled trial with follow-up survey and thematic analysis | | Traumatic hand injury (n=53) | Motivation  Visual aids  Perceived relationship | 100% of respondents felt that their appointment with their hand therapist was moderately to extremely important and 90% felt the appointment was moderate to extremely important in motivating them to do their exercises. Participants mentioned that desire or motivation to do exercises came from within and not from the DVD or brochure. No significant differences were found in the overall mean exercise compliance score between intervention and control groups (p=0.344). |
| ***Morrison (99s)*** | 2013 | Canada | Community | OT | To compare the contextual characteristics and experiences of the working alliance over the course of a community-based OT intervention by four dyads | Prospective study with multiple case study using 4 dyads and theoretical thematic analysis | | Multiple conditions (n=4)  Chronic pain following a motor vehicle accident (n=1) | Working alliance (Bond, Goals, Tasks)  Contextual (environmental) factors. | There are a variety of therapist, client, and environmental factors that interact with one another, each having a different impact on the process of alliance development. Therapist reflective self-development can enhance relational skills. Although therapists must take responsibility for creating a therapeutic atmosphere conducive to sound working alliance development, there are also elements having an impact on the alliance that are outside the therapist’s control. |
| ***Payton and Nelson (100s)*** | 1998 | USA | Primary care | PT | To discover how physical therapy patients, understand their role in therapy, particularly their role or involvement in goal-setting, treatment planning and evaluation of outcomes. | Descriptive study with qualitative element  Semi-structured tape recorded interviews  Content analysis | | Soft tissue/ orthopedic  (n=7) | Active involvement/  participation | Participation in assessing outcomes received the strongest support; participation in the evaluation of what helps therapeutically received somewhat less support; and patient involvement in goal- setting received the weakest support. Patients affirmed the importance of PT and developed a sense of personal relationship with their PTs. |
| ***Schoster et al (101s)*** | 2005 | USA | Community | PT | To assess participant satisfaction with the People with Arthritis Can Exercise (PACE) program and examine motivators and barriers to attending program classes. | Semi-structured interviews using constant comparison embedded in randomized controlled trial - | | Arthritis  (n=51) | Satisfaction  Motivators  Barriers | Participants’ high regard for their instructors increased desire to attend the class and facilitated safe engagement in the class exercises. Being nice, patient, friendly and polite are personalities and characteristics that increased perceived sense of support of PT |
| ***Stenmar and Nordholm (102s)*** | 1994 | Sweden | Primary care  Private practice  Rehabilitation  Occupational health | PT | To find out (1) what kinds of attributions physical therapists make regarding why PT works and (2) to what extent attributions are related to background variables (gender, age, education, experience, field of work). | Questionnaire survey and interviews | | PTs (n=140) | Patient-therapist interaction | Majority of the respondents believed that the patient's own resources and the patient-therapist relationship rather than the treatment techniques are the most important factors in explaining why PT works. |
| ***Van***  ***Puymbrouck (103s)*** | 2014 | USA | Post-acute rehabilitation | PT/OT | To determine if providing a client with a venue for goal identification, documentation, and maintenance might impact participation and satisfaction in a day rehabilitation setting | Quasi-experimental pilot study  Semi-structured and post intervention interviews | | Multiple conditions (n=20)  Arthritis/  orthopaedic injuries (n=3) | Goals  Satisfaction  Visual aids | Through an increased involvement of clients in the process of goal development and management, clients are able to better conceptualize the ongoing experiences of rehabilitation |
| **NARRATIVE/CRITICAL REVIEWS** | | | | | | | | | | |
| ***Bellner (104s)*** | 1999 | Sweden | NA | PT/OT | To delineate different senses of responsibility in the therapeutic relationship between OT and PT and their patients, in the context of ongoing professionalization. | Review article | | NA | Collaboration  Respect  Partnership | The interactive model concerns the relationship between therapist and patient in terms of a collaboration of equals making differing contributions. They have equal moral status in the relationship and owe each other equal respect, as they work together in decision making about treatment and other aspects of the relationship. |
| ***Barron et al (105s)*** | 2007 | UK | N/A | PT | To clarify the term patient and relate the concept to PT and highlight the impact that patients’ expectations may have on the outcome of PT and the implications for PT | Review | | MSK disorders | Expectation | PTs should consider patients’ expectations and related issues knowing how expectations may affect the progress and outcome of treatment |
| ***Clay and Hopps (106s)*** | 2003 | USA | N/A | PT/OT | To summarizes factors thought to affect adherence, including patient, treatment provider, environmental and treatment characteristics | Review | | NA | Adherence  Treatment accommodation | Various characteristics of service providers such as empathy,  Consistency, willingness to answer questions, scheduling follow-up appointments have demonstrated correlational relations with adherence. |
| ***Crandall et al (107s)*** | 2013 | USA | N/A | PT | To summarize a Cochrane review on exercise adherence interventions and present a clinical scenario based on a real patient to illustrate how the results of the review can be used to directly inform clinical decisions. | Discussion article | | Chronic knee osteoarthritis pain | Goal setting  Feedback  Visual aids  Knowledge exchange | Strategies such as providing additional educational material, goal setting, feedback, self-management practices may improve patient adherence to exercise. |
| ***Costa (108s)*** | 2008 | USA | NA | PT/OT | To discuss the difficult patient in rehabilitation and ways to improve the practitioner-client team. | Discussion paper | | General | Life experiences  Personal characteristics  Empathy  Active listening  Nonverbal skills | Maintaining a sense of humour, being as flexible as possible, communicating empathy, listening without interrupting, setting limits when necessary, and using self-reflection when conflict occurs are useful strategies for managing complex client-practitioner relationship. |
| ***Davis (109s)*** | 2009 | USA | NA | PT/OT | To discuss the role of empathy in clinical practice. | Review | | NA | Listening  Nonverbal skills  Environment  Attention | Within the clinical setting, empathy entails both cognitive and affective components and also behavioural component to communicate understanding to patients. Effective communication verbally and nonverbally is the means to expressing empathy in clinical practice. |
| ***Gorenberg and Taylor (110s)*** | 2013 | USA | N/A | OT | To introduce the Intentional Relationship Model (IRM) as a framework for teaching therapeutic use of self as a central component of the occupational therapy process. | Discussion paper | | N/A | Therapeutic use of self | Therapeutic use of self is an essential component of the occupational therapy process. The IRM provides a framework and common language that can be used in combination with instructional strategies to facilitate students’ learning of therapeutic relationship skills. |
| ***Hargreaves (111s)*** | 1982 | Australia | N/A | PT | To discuss the relevance of these skills and the rationale for their implementation in a professional setting. | Discussion | | MSK disorder | Nonverbal communication | Establishment of rapport may encourage the patient to express anxieties and fears without feeling the pressure of imminent dismissal.  Active participation by patients in the beginning of their therapy is a way to deal with the expectation of being a passive receiver. |
| ***Leach (112s)*** | 2005 | Australia | NA | PT/OT | To highlight the effects that strong therapeutic relationships can have on patient satisfaction, treatment compliance and client outcomes. | Discussion | | General | Trust  Communication  Rapport | The development of a strong therapeutic alliance and the subsequent production of positive client outcomes are dependent on effective communication skills, practitioner behaviour, collaboration, time and trust. |
| ***Lotze and Moseley (113s)*** | 2015 | Germany | N/A | PT | To argue that keen observation and open and respectful clinician-patient and scientist-clinician relationships have been critical for the emergence of effective rehabilitation approaches and will be critical for further improvements | Discussion | | Chronic pain | Safety in clinical practice | General aspects of interaction, such as being friendly and spending time concentrating on the patient’s needs, provide evidence of safety. Perception that the PT is informed, explaining pain, and explaining the rehabilitation approach in simple words also provide evidence of safety |
| ***McKenna and Mellson (114s)*** | 2013 | UK | N/A | OT | This opinion piece explores links between ability in emotional intelligence and the competent OT | Discussion | | N/A | Emotional intelligence (EI); | The therapeutic relationship with each service user is central, and EI abilities enable sensitivity in responding to, and in the understanding of, emotions, supporting effective emotional management. |
| ***Miciak et al (115s)*** | 2012 | Canada | N/A | PT | To explore the common factors model of psychotherapeutic intervention and discuss its relevance for PT practice | Critical review | | Acute and chronic low back pain | Contextual factors;  Expectation  Satisfaction  Beliefs  Therapeutic relationship  Provision of feedback | The model postulates that factors common across diverse interventions (nonspecific mechanisms) account for a larger component of treatment efficacy. Continued advance and evolution of the physical therapy profession requires creative and comprehensive analysis of all factors impacting clinical effectiveness. |
| ***Moffett and Richardson (116s)*** | 1997 | UK | N/A | PT | To review the evidence of the effects of the interaction between the PT and the patient | Critical review | | Chronic back pain | NA | Factors likely to affect the PT-patient relationship include well-directed communication, encouraging a patient’s sense of control, patient expectations, encouraging coping strategies, and reinforcing pain behaviour. |
| ***Neuman et al (117s)*** | 2009 | Germany | NA | PT/OT | To establish sound empirical evidence that empathy is a core element in the clinician-patient relationship with profound therapeutic potential. | Critical reviews | | General | Clinical empathy (CE) | CE causes emotional reactions among patients, such as feeling that one is being listened to, valued as an individual, understood and accepted and leads to short-term and/or intermediate outcomes such as satisfaction. |
| ***Podlog et al (118s)*** | 2014 | USA | N/A | PT | To discuss the psychological factors in sports injury rehabilitation and return to play | Narrative review | | Sport injury | Patient-practitioner relationship  Social factors | The 2 most influential social factors influencing athletes’ injury rehabilitation are the nature of patient-practitioner interactions and the effectiveness of social support provisions. |
| ***Radomski (119s)*** | 2011 | USA | N/A | OT | To bring the problem of non-adherence into the OT professional fold and clinical conversation. | Theoretical discussion | | Hand therapy | Advancing adherence to therapy recommendations | Building follow-up, support, and reinforcement can help clients adhere to long-term treatment recommendations that are typical of chronic health problems and develop new habits around recommended behaviours, regimens or activities. This in-depth consideration of a multistep process nested in an ecological model suggests that adherence involves patients and OTs collaborating as equals, establishing agreement on a proposed course of action and together trying to anticipate the kinds of supports that will enable patients to adhere to therapy to meet their own goals |
| ***Spetch and Kolt (120s)*** | 2011 | Australia | N/A | PT | To critically evaluate the adherence literature related to sport injury rehabilitation | Critical review | | Sport Injury | Adherence  Predictors  Theoretical considerations  Strategies | Athletes who feel that the PT treating their injury is honest, genuinely interested in their well-being, and aware of any psychological manifestations relating to their injury are more motivated to adhere to their rehabilitation program  Working together to establish and achieve challenging yet realistic and positive goals promote adherence to rehabilitation programmes. |
| ***Smith-Gabai (121s)*** | 2007 | USA | N/A | OT | To provide an expert’s perspective on client empowerment | Expert opinion | | N/A | Client empowerment | Assisting clients in developing problem-solving skills, increasing their critical awareness of their strengths and barriers, and helping them generate their own strategies and solutions will foster their empowerment and help them to achieve their stated health care goals. |
| ***Szybek et al (122s)*** | 2000 | Sweden | N/A | PT | To elaborate the concept of the physiotherapist–patient relationship, taking counselling and psychotherapeutic encounters as the model. | Critical review | | General | Level of transference | Insight-oriented PT should be seen as implementing a patient-oriented perspective with the limited aim of facilitating successful physiotherapeutic treatment by way of improving the working relationship. |
| ***Tickle-Degnen (123s)*** | 2002 | USA | N/A | OT | To offer suggestions for using research evidence in the daily practice of client-centred OT through a model of effective interpersonal relationship | Expert opinion | | N/R | Client-centred practice  Therapeutic relatisonship | The partnership of client-centred OT requires an ongoing communication process that involves the exchange of information used to help choose therapeutic tasks and goals, regulate emotion and bond ﬂuctuations that emerge in response to challenging therapeutic tasks, and to support ﬂexible adaptation by therapist and client to the evolving responses and goals of the client during therapy. |
| ***Verkaaik et al (124s)*** | 2010 | New Zealand | N/A | PT/OT | To examines the role of power distribution in partnerships between health consumers and professionals in determining successful desired outcomes, and the contributing role of consumer knowledge and autonomy. | Conceptual analysis | | Post-acute rehabilitation | Power distribution | The P2 framework will lead to stronger outcomes through the provision of genuine choice in patient-therapist interaction and power sharing. The P2- framework allows therapists to measure early alliance from increased health consumer knowledge and autonomy. |
| ***Vowles and Thompson (125s)*** | 2012 | UK | N/A | PT/OT | To review the literature on patient-provider relationship and make recommendations for future clinical and academic work. | Critical review | | Chronic pain | Empathy  Positive regard  Congruence and genuine  Client feedback  Goal consensus  Collaboration  Group cohesion | Essentially, there is evidence to suggest that positive response to  treatment depends at least as much on the relationship between patient and provider as it does on the technical  aspects of the intervention itself. |
| ***Walker (126s)*** | 1995 | UK | N/A | PT | To consider how patient compliance and possibly the placebo effect may play a much greater part than is often appreciated in securing a truly successful episode of care. | Critical review | | General including orthopedics | Trust  Confidence  Interest  Concern  Knowledge exchange | When PTs gain the patient’s confidence and trust, they become amenable to the therapeutic intervention and demonstrate better compliance. The quality of the relationship is the main source of information available to the patient about PT skill. |
| ***William and Harrison (127s)*** | 1999 | UK | N/A | PT | To explore the power dynamic in PT interaction | Critical review | | General | Interpersonal power  Power exertion  Power differences | The evidence reveals a diversity of power manifestations constructing the framework of the therapeutic relationship which exposes a complex interplay of patient, physiotherapist and external environmental variables. There is a dialectic of control whereby both sides of the dyad have access to power sources. |
| **SYSTEMATIC REVIEWS** | | | | | | | | | | |
| ***Besley et al (128s)*** | 2010 | New Zealand | NA | PT | To explore existing literature regarding the ‘therapeutic relationship’ in physiotherapy to identify  what are considered the core components and critically appraise the conceptual basis of two commonly used measures of the  Working Alliance Inventory and the Helping Alliance Questionnaire Version Two. | Systematic review | 16 studies  4 MSK studies | | Therapeutic relationship | Eight key themes were identified including: patient expectations; personalised therapy; partnership; physiotherapist roles and responsibilities; congruence; communication; relationship/  relational aspects; and influencing factors. Each of the measures addressed some components of therapeutic relationship well  (namely congruence, partnership, and physiotherapists roles and responsibilities), while some components were poorly addressed  (communication, personalised therapy, and relational aspects). |
| ***Hall et al (129s)*** | 2010 | Australia | NA | PT | To identify and summarize studies that have used and analyzed the alliance as a predictor of outcome and adherence in physical rehabilitation settings and to determine whether there is an association between the alliance and the treatment outcome of physical rehabilitation programs. We hypothesized that the patient-therapist alliance would have a positive correlation with  treatment outcome. | Systematic review | 13 studies  6 MSK studies | | Communication  - Verbal behaviors  - Nonverbal cues  Satisfaction  Working Alliance | TA was positively related to treatment satisfaction in patients with MSK conditions.  Significant positive associations  were found between the alliance  and the patient’s global perceived  effect of treatment, change in pain, physical function, patient satisfaction with treatment, depression, and general  health status. |
| ***O’Keeffe et al (130s)*** | 2016 | Ireland | NA | PT | To systematically investigate physical therapists’ and patients’ perceptions of factors that inﬂuence patient-therapist interactions in musculoskeletal settings. | Qualitative systematic review and meta-synthesis | 13 studies (MSK) | | Four themes were perceived to inﬂuence patient-therapist interactions: (1) physical therapist interpersonal and communication skills (presence of skills such as listening, encouragement, conﬁdence, being empathetic and friendly, and nonverbal communication), (2) physical therapist practical skills (physical therapist expertise and level of training, although the ability to provide good education was considered as important only by patients), (3) individualized patient-centered care (individualizing the treatment to the patient and taking patient’s opinions into account), and (4) organizational and environmental factors (i.e. time and ﬂexibility with care and appointments). | A mix of interpersonal, clinical, and organizational factors are perceived to inﬂuence patient-therapist interactions. |

**Reference List of Articles Included in the Scoping Review**

1s. Adamson BJ, Sinclair-Legge G, Cusick A and Nordholm L, Attitudes, values and orientation

to professional practice: a study of Australian Occupational Therapists, Bri J Occup Ther,

1994;57:476-80.

2s. Ambady N, Koo J, Rosenthal R, Winograd CH. Physical therapists’ nonverbal

communication predicts geriatric patients’ health outcomes. Psychol Aging. 2002;17: 443–

452. 28

3s. Azoulay L, Ehrmann-Feldman D, Truchon M, Rossignol M. effects of patient-clinician

disagreement in occupational low back pain: a pilot study. Disabil and Rehabil. 2005;27:817-

23.

4s. Baker SM, Marshak HH, Rice GT, Zimmerman GJ, Patient participation in physical therapy

goal setting, Phys Ther. 2001;81:1118-1126.

5s. Bassett S, Petrie KJ, The effect of treatment goals on patient compliance with physiotherapy

exercise programmes; Physiother, 1999;85:130-37.

6s. Beattie P, Turner C, Dowda M, Dowda M, Michener L, Nelson R. The MedRisk Instrument

for Measuring Patient Satisfaction with Physical Therapy Care: a psychometric analysis. J

Orthop Sports Phys Ther. 2005;35:24–32

7s. Besley J, Kayes NM, McPherson KM, Assessing the measurement properties of two

commonly used measures of therapeutic relationship in physiotherapy, New Z J Physioth,

2010;39:75-80.

8s. Bliss EL. The roles of attachment, depression, and the working alliance in predicting

treatment outcomes in chronic pain patients seeking physical therapy services, Dissertation

Abstracts International: Section B: The Sciences and Engineering, University of Miami,

2010,71(1-B), 271

9s. Burns JW, Higdon LJ, Mullen JT, Lansky D, Wei JM, Relationships among patient hostility,

anger expression, depression and the working alliance in a work hardening program; Annals

Behav Med, 1999;27:77-82.

10s. Chan DK, Lonsdale C, Ho P, Yung PS, Chan KM, Patient motivation and adherence to

postsurgery rehabilitation exercise recommendations: the influence of physiotherapists’

autonomy-supportive behaviors, Arch Phys Med Rehabil, 2009;90:1977-82

11s. Chan D, Can F, Patients’ adherence/compliance to physical therapy home exercises,

Fizyote Rehabil. 2010;21:132-39

12s. Cheing GLY, Lai AKM, Vong SKS, Chan FH, Factorial structure of the pain rehabilitation

expectations scale: a preliminary study, Inter J Rehabil Res, 2010;38:88-94.

13s. Chen CY, Neufeld PS, Feely CA, Skinner CS, Factors influencing compliance with home

exercise programs among patients with upper extremity impairment; Am J Occup Ther,

1999;53:171-80.

14s. Cole MB, McLean V, Therapeutic relationship re-defined, Occup Ther Mental Health,

2003;19:33-56

15s. Crooks V, Kingsbury P, Snyder J, Johnston R. What is known about the patient’s experience

of medical tourism? A scoping review. BMC Health Ser Res, 2010;10:266

16s. do Nascimento PR, Ferreira PH, Azevedo FM, Filho RFN, Relationship between therapeutic

alliance and deep abdominal muscle recruitment in nonspecific low back pain sufferers,

Fisoter Pesq 2014;21:320-326.

17s. Eklund M, Erlandsson L-K, Wastberg BA, A longitudinal study of the working relationship

and return to work: perceptions by clients and occupational therapists in primary health

care, BMC Fam Pract, 2015;16:46

18s. Ferreira PH, Ferreira ML, Maher CG, et al. The therapeutic alliance between

Physiotherapists and patients predicts outcome in chronic low back pain. Phys Ther,

2013;93:470-78.

19s. Foster NE, Thomas E, hill JC, Hay EM. The relationship between patient and practitioner

expectations and preferences and clinical outcomes in a trial of exercise and acupuncture for

knee osteoarthritis. Eur J Pain, 2010;14:402-9

20s. Fuentes J, Armijo-Olivo S, Funabashi M, Miciak M, Dick B, Warren S, Rashiq S, Magee

DJ, Gross DP. Enhanced therapeutic alliance modulates pain intensity and muscle pain

sensitivity in patients with chronic low back pain: an experimental controlled study. Phys

Ther, 2014;94:477-89

21s. Gleeson R, Chant A-M, Cusick A, Hodgers E, Non-compliance with occupational therapy

outpatient attendance: a quality assurance study, Aust Occup Ther J 1991;55-61

22s. Grannis CJ. The ideal physical therapists as perceived by the elderly patient, Phys

Ther, 1981;61:479-86.

23s. Hall AM, Ferreira ML, Clemson L, Ferreira P, Latimer J, Maher CG, Assessment of the

therapeutic alliance in physical rehabilitation: a RASCH analysis, Dis & Rehab,

2012;34:257-66.

24s. Hills R, Kitchen S, Satisfaction with outpatient physiotherapy: A survey comparing the

views of patients with acute and chronic musculoskeletal conditions, Physioth Theory Pract,

2007;23:21-36.

25s. Jackson B, Dimmock JA, Taylor IM, Hagger MS, The Tripartite Efficacy Framework in

client-therapist rehabilitation interactions: Implications for relationship quality and client

engagement, Rehab Psychol, 2012;57:308-19.

26s. Jensen GM, Lorish CD. Promoting patient cooperation with exercise programs; linking

research, theory and practice, Arth Care & Res, 1994,7:181-89

27s. Kersten P, White PJ, Tennant A, The consultation and relational empathy measure: an

investigation of its scaling structure, Disabil & Rehabil, 2012;34:503-9.

28s. Kerssens JJ, Sluijs EM, Verhaak PFM, Knibble HJ, Hermans IMJ. Educating patient

educators: enhancing instructional effectiveness in physical therapy for low back pain

patients, Patient Edu & Couns 1999;37:165-76.

29s. Knight PK, Cheng A N-J, Lee GM, Results of a survey of client satisfaction with outpatient

physiotherapy care, Physio, Theory and Pract, 2010;26:297-307.

30s. Levy AR, Polman RC, Borkoles E, Examining the relationship between perceived autonomy

support and age in the context of rehabilitation adherence in sport, Rehabil Psychol,

2008;53:224-30.

31s. Lysack C, Dama M, Neufeld S, Andreassi E, 2005, Compliance and satisfaction with home

exercise: a comparison of computer-assisted video instruction and routine rehabilitation

practice, J Allied Health, 2005;3:76-82.

32s. Medina-Mirapeix F, del Bano-Aledo Me, Martinez-Paya JJ, Lillo-Navarro MC and Escolar-

Reina P, Development and validity of the questionnaire of patients’ experiences in post-

acute outpatient physical therapy settings, Phys Ther, 2015;95:767-77.

33s. Murray A, Hall AM, Williams GC, McDonough SM, Ntoumanis N, Taylor IM et al, effect

of a self-determination theory –based communication skills training program on

Physiotherapists’ psychological support for their patients with chronic low back pain: a

randomized controlled trial, Archives of Physical Medicine and Rehabilitation, 2015; article

in press.

34s. Niedermann K, de Bie RA, Kubli R, Ciurea A, Steuree-Stey C, Villiger PM, Buchi S.

Effectiveness of individual resource-oriented joint protection education in people with

rheumatoid arthritis. A randomized controlled trial, Patient Education and Counseling,

2011;82:42-48.

35s. Osmotherly P and Higginbotham N, assessing patient intention to perform a home-based

exercise program for back and shoulder pain, Physio Ther & Pract, 2004;20:57-71.

36s. Roberts L and Bucksey SJ. Communicating with patients: what happens in practice?

Phys Ther, 2007; 87:586-94.

37s. Sluijs EM, A checklist to assess patient education in physical therapy practice: development

and reliability, Phys Ther, 1991;71:561-69.

38s. Sluijs EM Kok GJ, van der Zee J. Correlates of exercise compliance in physical therapy

Phys Ther. 1993;73:771–82.

39s. Taylor RR, Lee S W, Kielhofner G, Practitioners’ use of interpersonal modes within the

therapeutic relationship: Results of a nationwide survey, OTJR: Occup, Part & Health,

2011;31:6-14.

40s. Thomson D, Hassenkamp A-M, Mansbridge C, The measurement of empathy in a clinical

and non-clinical setting, Physioth, 1997;83:173-80

41s. Tousignant M, Boissy P, Moffet H, Corriveau H, Cabana F, Marquis, F et al, Patients'

satisfaction of healthcare services and perception with in-home telerehabilitation and

physiotherapists' satisfaction toward technology for post-knee arthroplasty: An embedded

study in a randomized trial, Telemed & e-Health, 2011;17:376-82.

42s. Vong SK, Cheing GL, Chan F, So EM, Chan CC, Motivational Enhancement Therapy in

addition to Physical Therapy improves motivational factors and treatment outcomes in

people with low back pain: a randomized controlled trial, Arch Phys Med Rehabil,

2011;92:176-83

43s. Wright BJ, Galtieri NJ, Fell M, Non-adherence to prescribed home rehabilitation exercises

for musculoskeletal injuries: the role of the patient-practitioner relationship, Journal of

Rehabilitation Medicine, 2013;46:00-00 Epub ahead of print.

44s. Aguilar A, Stupans L, Scutter S, King S, Exploring the professional values of Australian

physiotherapists. Physiother Res Inter, 2012;18:27-36.

45s. Bamford R and Walker D-M, A qualitative investigation into the rehabilitation experience

of patients following wrist fracture; Hand Therapy, 2010;15:54-61.

46s. Bassett SF and Tango SM, Maori people’s experiences of being physiotherapy patients: a

phenomenological study, New Z J of Physio, 2002;30:30-40.

47s. Bunzli S, McEvoy S, Dankaerts W, O’Sullivan P, O’Sullivan K, Patient perspectives on

participation in cognitive functional therapy for chronic low back pain, Phys Ther.

2016;96:1397-1407.

48s. Campbell R, Evans M, Tucker M, Quilty B, Dieppe P, Donovan JL. Why don’t patients do

their exercises? Understanding non-compliance with physiotherapy in patients with

osteoarthritis of the knee, J Epid &Cl Com Health, 2001;55:132-138.

49s. Cipriani J, Hess S, Higgins H, Resavy D, Sheon S, Szychowski M, Holm MB, Collaboration

in the therapeutic process: older adult’s perspectives, Phys & Occup Ther Geriat.

1999;17:43-54.

50s. Cooper K, Smith BH, Hancock E, Patient-centredness in physiotherapy from the perspective

of the chronic low back pain patient, Physiother, 2008;94:244-52

51s. Coutu MF, Baril R, Durand MJ, Cote D, Cadieux G, Clinician-patient agreement about the

work disability problem of patients having persistent pain: why it matters, J Occup Rehabil,

2013;23:82-92

52s. Crepeau EB, Garren KR, I looked to her as a guide: the therapeutic relationship in hand

therapy. Disabil & Rehabil, 2011;33:872-81.

53s. Dahlgren MA, Amlquist A, Krrok J, Physiotherapists in Balint group training,

Physiothe Res Intern, 2000;5:85-96.

54s. Del Bano-Aledo M, Medina-Mirapiex F, Escolar-Reina P, Montilla-Herrador J, Collins SM,

Relevant patient perceptions and experiences for evaluating quality of interaction with

physiotherapists during outpatient rehabilitation: a qualitative study, Physiother.

2014;100:73-79.

55s. Ekerholt K, Bergland A, The first encounter with Norwegian psychomotor physiotherapy:

patients’ experiences, a basis for knowledge, Scandinavian Journal of Public Health,

2004;32:403-410.

56s. Escolar-Reina P, Medina-Mirapeix F, Gascon-Canovas JJ, Montilla-Herrador J, Jimeno-

Serrano F, Sousa SLD et al. How do care-provider and home exercise program

characteristics affect patient adherence in chronic neck and back pain: a qualitative study,

BMC Health Serv Res, 2010;10:60.

57s. Farin E, Gramm L, Kosiol D, Development of a questionnaire to assess communication

preferences of patients with chronic illness, Patient Educ & Couns, 2011;82:81-88.

58s. Gard G, Gyllensten AL, Salford E, Ekdahl C, Physical therapist’s emotional expressions in

interviews about factors important for interaction with patients, Physiother, 2000;86:229-40.

59s. Greenfield BH, The meaning of caring in five experienced physical therapists,

Physiother Theory & Pract, 2006;22:175-87.

60s. Greenfield BH, Anderson A, Cox B, Tanner MC, Meaning of caring to 7 novice physical

therapists during their year of clinical practice, Phys Ther, 2008;88:1154-66.

61s. Gyllensten AL, Gard G, Salford E, Ekdahl C, Interaction between patient and

physiotherapist: a qualitative study reflecting the physiotherapist’s perspective, Physio Res

Int. 1999;4:89-109

62s. Harrison, K. and Williams, S, Exploring the power balance in physiotherapy. J of Ther &

Rehab, 2000;7: 355-61.

63s. Harman K, MacRae M, Vallis M, Bassett R. Working with people to make changes: a

behavioural change approach used in chronic low back pain rehabilitation, Physiother Can,

2014;66:82-90.

64s. Haywood H, Pain H, Ryan S, Adams J. Continuing professional development: issues raised

by nurses and allied health professionals working in musculoskeletal settings,

Musc Care, 2013;11:136-44.

65s. Hinman RS, Delany CM, Campbell PK, Gale J, Bennell KL, Physical therapists, telephone

coaches, and patients with knee osteoarthritis: qualitative study about working together to

promote exercise adherence, Phys Ther, 2015;95

66s. Hurley MV, Walsh N, Bhavnani N, Britten N, Stevenson F, Health beliefs before and after

participation on an exercise-based rehabilitation programme for chronic knee pain: Doing is

believing, BMC Musc Dis, 2010;11:31

67s. Karnad P, McLean S, Physiotherapists’ perceptions of patient adherence to home exercises

in chronic musculoskeletal conditions, Inter J Physiother & Rehabil, 2011;1-14

68s. Kidd MO, Bond CH, Bell ML, Patients’ perspectives of patient-centredness as important in

musculoskeletal physiotherapy interactions: a qualitative study, Physiother,

2011;97:154-62.

69s. Kumlin WI, Kroksmark T. The first encounter – Physiotherapists’ conceptions of

establishing therapeutic relationships, Scand J Caring, 1992;6:37-44.

70s. Larsson I, Liljedahl K, Gard G. Physiotherapist’s experience of client participation in

physiotherapy interventions: a phenomenological study, Adv Physiother, 2010;12:217-23.

71s. Liddle SD, Baxter GD, Gracey JH, Chronic low back pain: patients’ experiences, opinions

and expectations for clinical management, Disabil &Rehabil, 2007;29:1899-1909.

72s. Littlewood C, Milliaras P, Mawson S, May S, Walters S, Patients with rotator cuff

tendinopathy can successfully self-manage, but with certain caveats: a qualitative study,

Physiother, 2014;100:80-85.

73s. Mann C and Cooberman-Hill R, Health care provision for osteoarthritis: concordance

between what patients would like and what health professionals think they should have.

Arth Care & Res, 2011;63:963-72.

74s. May SJ, Patient satisfaction with management of back pain, (Part 1), An explorative,

qualitative study into patients' satisfaction with physiotherapy, (Part 2), Physiother,

2001;87:4-2

75s. Medina-Mirapeix F, Del Bano Aledo, Oliviera-Sousa SL, Escola-Reina P, Collins

SM, How the rehabilitation environment influences patient perception of service quality: a

qualitative study, Arch Phys Med Rehab, 2013;94:112-7.

76s. Norby E, Bellner AL. The helping encounter, Scand J Caring Sciences, 1995;9:41-46.

77s. 130. Øien AM, Steihaug S, Iversen S, Raheim M, Communication as negotiation processes in long-

Term physiotherapy: a qualitative study. Scand J Caring Sci, 2011;25:53-61.

78s. Owen OG, Goodge P, Physiotherapists talking to patients, Patient Counsel & Health

Edu. 1981;3:100-102.

79s. Palmadottir G, Client-therapist relationships: experiences of occupational therapy clients in

rehabilitation, Bri J Occup Ther, 2006;69:394-401.

80s. Papi E, Murtagh GM, McGregor AH, Wearable technologies in osteoarthritis: a qualitative

study of clinicians’ preferences, Bri Med J Open, 2016;16:6e009544.

81s. Peiris CL, Taylor NF, Shields, Patients value patient-therapist interactions more than the

amount or content of therapy during inpatient rehabilitation: a qualitative study, J Physiother

2012;58:261-68

82s. Petursdottir U, Arnadottir S A, Halldorsdottir S, Facilitators and barriers to exercising

among people with osteoarthritis: a phenomenological study, Phys Ther, 2010;90:1014-

25.

83s. Potter M, Gordon S, and Hamer P, The difficult patient in private practice physiotherapy: a

qualitative study, Aus J Physiother, 2003(a);49:53-61.

84s. Potter M, Gordon S, Hamer P, The physiotherapy experience in private practice: the

patients’ perspective, Aus J Physiother, 2003(b);49:195-202

85s. Rosa SA, Hasselkus BR, Connecting with patients: the personal experience of professional

helping, The Occup Ther J Res, 1996;16:245-60.

86s. Rosa, S. A., & Hasselkus, B. R. Finding common ground with patients: The centrality of

compatibility. Am J Occup Ther, 2005;59, 198–208

87s. Slade SC, Molloy E, Keating JL, People with chronic low back pain who have participated

in exercise programs have preferences about exercise: a qualitative study, Aus J Physiother,

2009;55: 115–21.

88s. Stenner R, Swinkels A, Mitchell T, Palmer S, Exercise prescription for patients with non-

specific chronic low back pain: a qualitative exploration of decision making in

physiotherapy practice, Physiother, 2016; 102:332-38.

89s. Swardh E, Biguet G, Opava CH, Views on exercise maintenance: variations among patients

with rheumatoid arthritis. Phys Ther. 2008;88:1049-60.

90s. Thomson D, An ethnographic study of physiotherapists’ perceptions of their interactions

with patients on a chronic pain unit, Physiother Theory & Pract, 2008;24:408-22.

91s. Thornquist E, Examination and communication: a study of first encounters between patients

and physiotherapists, Fam Pract, 1992;9:195-202.

92s. Veenhof C, van Hasselt TJ, Koke AJA, Dekker J, Bijlsma JWJ, van den Ende CHM, Active

involvement and long-term goals influence long-term adherence to behavioural graded

activity in patients with osteoarthritis: a qualitative study, Aus J Physiother, 2006;52:273-78.

93s. Wikman AM, Falthom, Patient empowerment in rehabilitation: “somebody told me to get

rehabilitated” Adv Physiother, 2006;8:23-32.

94s. Waters S, Edmondston SJ, Yates PJ, Gucciardi DF, Identification of factors influencing

patient satisfaction with orthopaedic clinic consultation: a qualitative study, Man Ther,

2016;25:48-55

95s. Wilson S, Chaloner N, Osborn M, Gauntlett-Gilbert J, Psychologically informed

physiotherapy for chronic pain: patient experiences of treatment and therapeutic process,

Physiother, 2017;103:98-105

96s. Favre C, Deriaz O, Hanon R, Luthi F, Care related pain in rehabilitation after orthopedic

trauma: an exploratory study with qualitative data, Annals Phys & Rehabil Med,

2015;58:132-38.

97s. Freene N, Waddington G, Chesworth W, Davey R, Cochranes T, Community group

exercise versus physiotherapist-led home-based physical activity program: barriers, enablers

and preferences in middle-aged adults, Physiother, Theory & Pract, 2014;30:85-93.

98s. Kingston GA, Williams G, Gray MA, Judd J. Does a DVD improve compliance with home

exercise programs for people who have sustained a traumatic hand injury? Results of a

feasibility study, Disabil & Rehabil, 2014;9:188-194

99s. Morrison T, Individual and environmental implications of working alliances in occupational

therapy, Bri J Occup Ther, 2013;76:507-14.

100s. Payton OD and Nelson CE, A preliminary study of patients’ perceptions of certain aspects

of their physical therapy experience, Physiother, Theory & Practice, 1996; 12:27-38.

101s. Schoster B, Callahan LF, Meier A, Mielenz T, DiWalker O L, Prev Chronic Dis,

2005;2:A11

102s. Stenmar L, Nordholm L, Swedish physical therapists’ beliefs on what makes therapy work,

Phys Ther, 1994;74:1034-39.

103s. Van Puymbrouck, Promoting client goal ownership in clinical practice, Open J Occup

Ther, 2014;2

104s. Bellner AL, Senses of responsibility: a challenge for occupational and physical therapists

in the context of ongoing professionalization, Scand Journal Caring Sci, 1999;13:55-62

105s. Barron CJ, Klaber Moffett JA, Potter M, Patient expectations of physiotherapy: definitions,

concepts, and theories, Phys Theory and Pract, 2007;23:37-46.

106s. Clay DL and Hopps JA, Treatment adherence in rehabilitation: the role of treatment

accommodation, Rehab Psychol, 2003;48:215-19.

107s. Crandall S, Howlett S, Keysor JJ, Exercise adherence interventions for adults with chronic

musculoskeletal pain, Phys Ther, 2013;93:17-21.

108s. Costa DM, Working with the difficult client, Occupational Therapy Practice, 2008;7:15-18

109s. Davis MA, A perspective on cultivating clinical empathy, Comp Ther in Clin Pract,

2009;15:76-79.

110s. Gorenberg M, Instructional insights: Continuing professional education to enhance

therapeutic relationships in occupational therapy, Occup Ther in Health Care,

2013;27:393-98.

111s. Hargreaves S, The relevance of non-verbal skills in physiotherapy, Aus J Physiother,

1982;28:19-22.

112s.Leach MJ, Rapport: A key to treatment success, Comp Ther in Clin Pract, 2005;11:262-65.

113s. Lotze M and Moseley GL, Theoretical considerations for chronic pain rehabilitation, Phys

Ther, 2015;95:131620. 114s. McKenna J, Mellson J, Emotional intelligence and the occupational therapist, Bri J

Occup Ther, 2013;76:427-30.

115s. Miciak M, Gross DP, Joyce A, A review of the psychotherapeutic ‘common factors’ model

and its application in physical therapy: the need to consider general effects in physical

therapy practice, Scand J Caring Sci, 2012;26:394-403.

116s. Moffett JA, Richardson PH, The influence of the physiotherapist-patient relationship on

pain and disability, Physiother, Theory & Pract, 1997;13:89-96.

117s. Neumann M, Bensing J, Merser S, Ernstmann N, Ommen O, Pfaff H, Analyzing the

“nature” and “specific effectiveness” of clinical empathy: a theoretical overview and

contribution towards a theory-based research agenda, Patient Education and Counselling,

2009;24:339-46.

118s. Podlog L, Heil J, Schulte S, Psychological factors in sports injury rehabilitation and return

to play, Phys Med & Rehab Clinics of North America, 2014;25:915-30.

119s. Radomski MV. More than good intentions: advancing adherence to therapy

recommendations, Am J Occup Ther, 2011;65:471-77.

120s. Spetch LA, Kolt GS, Adherence to sport injury rehabilitation: implications for sports

medicine providers and researchers, Phys Ther Sport, 2001;2:80-90

121s. Smith-Gabai H, Client empowerment, Occup Ther Practice, 2007;23-25.

122s. Szybek K, Gad G, Linden. The physiotherapist-patient relationship: applying a

psychotherapy model, Physiother Theory & Pract, 2000;16:181-93.

123s. Tickle-Degnen L, Client-centred practice, therapeutic relationship and the use of research

evidence Am J Occup Ther, 2002;56:470-74

124s. Verkaaik J, Sinott AK, Cassidy B, Freeman C, Kunowski T, The productive partnerships

framework: harnessing health consumer knowledge and autonomy to create and predict

successful rehabilitation outcomes; Dis & Rehabil, 2010;32:978-85.

125s. Vowles KE, Thompson M, The patient-provider relationship in chronic pain, Current Pain

& Headache Reports, 2012;16:133-38.

126s. Walker A, Patient compliance and the placebo effect, Physiotherapy, 1995;81:120-126.

127s. William S, Harrison K, Physiotherapeutic interactions: a review of the power dynamic,

Phys Ther Rev, 1999;4:37-50.

128s. Besley J, Kayes NM, McPherson KM, Assessing therapeutic relationships in

physiotherapy: literature review. New Z J Physioth. 2010;39:81-91

129s. Hall AM, Ferreira PH, Maher CG, Latimer J, Ferreira ML, The influence of the therapist-

patient relationship on treatment outcome in physical rehabilitation: a systematic review,

Phys Ther, 2010;90:1099-110.

130s. O’Keeffe M, Cullinane PC, Hurley J, Leahy I, Bunzli S, O’Sullivan PB, O’Sullivan K.

What influences patient-therapist interactions in musculoskeletal physical therapy?

Qualitative systematic review and meta-synthesis, Phys Ther, 2016;96:609-22.
