# Supplementary material for: Characteristics of therapeutic alliance in musculoskeletal physiotherapy and occupational therapy practice: a scoping review of the literature
Source: BMC Health Serv Res. 2017 May 30;17:375. doi: 10.1186/s12913-017-2311-3 (PMC5450083; doi:10.1186/s12913-017-2311-3)
Supplement: Supplementary file 2 — Therapeutic Alliance Terms. Description of therapeutic alliance terms detailed in included studies. (DOCX 63 kb) [file 12913_2017_2311_MOESM2_ESM.docx]

**Additional file 2:** Abstracted findings from coding therapeutic alliance themes in qualitative studies

| **Overarching**  **Themes** | **Key Constructs** | **Summary of Abstracted Findings** | **Reference No (s)**  **Identifying**  **Abstracted**  **Findings** |
| --- | --- | --- | --- |
| **Communication** | Verbal and nonverbal skills | *Patient perspective:*   - Good communication involves ‘taking time over explanations, using appropriate terminology; really listening, understanding and getting to know the patient, encouraging the patient’s participation in the communication process’ and appropriate body and facial expressions. - Ordinary conversations serve as a foundation for trust and affirms the social connection. - Service quality is judged by therapist’s attitudes and behaviors when providing information and education. - Two-way information reassures and informs the patient consists of good listening, skills, paraphrasing and explaining, reassurance about pain, and correct and understandable interpretation of lay speech. - A good therapist provides feedback on a visit-by-visit basis. - Good communication help patients know what to expect during care and leave them with a positive attitude. - Confidence is built with clear communication – ‘because she talked to me about what we’re going to do and why we’re doing it”   *Professional perspective:*   - Communication inspires, empowers and motivates. Patients. A good communicator is ‘open’, ‘respected views’ and ‘listened’. - Reciprocity of ordinary conversations create an atmosphere that enables joint problem solving is essential for effective therapy. - Communication needs to be collaborative, patient-centred and consistent for integrated care to be effective. It helps to understand patient expectations and problems. - Active listening skills include eye contact, nodding the head and maintaining an open posture. - Dissatisfaction with service results when patients feel they haven’t been listed to.   *Observer perspective:*   - Communication should be used to enter more fully into the patient’s situation by focusing in the key needs or problems as perceived by the patient. - Constructive feedback involves giving praise or helpful feedback on performance and progress. - Openness to exploring ways of working through challenges; communication barriers, lack of interest and difference of opinions, result in satisfying experiences feeling of closeness to patients involved. - The therapist and patient’s nonverbal and/or verbal communication are initiatives to renegotiate the relationship. It is impossible to not communicate because all behavior including speech, tone of voice, silence, withdrawal, immobility or denial is communication. | Cooper et al (50s),  Gyllensten et al (61s)  Crepeau and Garren (52s)  Del Bano-Aledo et al (54s)  Kidd et al (68s), Petursdottir et al (81s)  Potter et al (83s)  Waters et al (93s)  Morrison (98s)  Aguilar et al (44s)  Crepeau and Garren (52s)  Hinman et al (65s)  Karnad & McLean (67s)  Potter et al (82s)  Waters et al (93s)  Norby et al (75s)  Owen & Goode (77s)  Rosa & Hasselkus (85s)  Øien () |
|  | Visual aids | *Patient perspective:*   - Patients believe that use of diagrams and additional written instructions are the best methods of communication. - A good therapist uses visual aids and gives written information to help patient understand the problem and treatment.   *Professional perspective:*   - Wearing technologies monitoring patients would enhance rather than interfere with the professional-patient relationship - Visual aids can assist with explanations | Bassett and Tango (46s)  Potter et al (83s)  Papi et al (79s)  Potter et al (82s) |
| **Congruence** | Agreement on goals and tasks | *Patient perspective:*   - The roles of the participants in therapy was highly interactive and based on sharing of information throughout the treatment. “I was asked if I like idea of this treatment and if I could cope with it…especially the home exercises and clinic attendance”. - Agreement between proposed strategy and personal thoughts, beliefs and thoughts associated with perceived cause, course, immediate and long-term consequences and control over illness are crucial. - Positive outcomes are desirable when improvement is communicated and measurable. - Patients value opportunities to set the pace and identify therapy goals with considerable initiative and responsibility. - Coalition involves working together towards a clear goal, solving issues of importance to both patient and therapist and formally distributing tasks between both parties. This is based on high level of confidence and awareness of worth and either’s ability.   *Professional perspective:*   - Proper identification of illness representation helps to formulate specific, concrete goals. - Establishing cooperation, having a contract, a goal and agreement about treatment. - Establishing treatment goals and solving problems.   Observer perspective:   - Therapist ability to listen attentively to the patient and negotiate a result leaves both parties empowered. | Bassett and Tango (46s)  Coutu et al (51s)  Kidd et al (68s)  Palmadottir (78s)  Palmadottir (78s)  Coutu et al (51s)  Gyllensten et al (61s)  Rosa et al (84s)  Thomson (89s) |
|  | Problem identification | *Patient perspective:*   - Clear explanation about the injury and reasons for treatment are important - Receiving information, practical advice and opportunity to discuss concerning issues with therapists help appreciate problems and how to address them - Tackling the problem with what works is crucial to the relationship   *Professional perspective:*   - Being able to evaluate what the patient perceives as the problem is key. | Bassett and Tango (46s)  Hurley et al (66s)  May (74s)  Gyllensten et al (61s) |
| **Connectedness** | Friendliness | *Patient perspective:*   - Therapists that are friendly, knowledgeable and compassionate garnered a positive attitude from patients. - Participant’s high regard for professional’s niceness, patience, friendliness, and politeness, increased desire to engage in program.   *Professional perspective:* | Peiris et al (80s)  Schoster et al (100s) |
|  | Perceived good relationship | *Patient perspective:*   - Patients like to consult therapists who are caring, friendly and pleasant. - Fellowship is described as a strong sense of connection due to interactions that are relaxed and natural, based on mutual dignity, collective decision making, involvement and sharing of personal experiences. - Patients tend to focus on the personal attributes of current and subsequent interactions and not the content or outcomes of physiotherapy rehabilitation. - Relatedness is more like a face to face thing and you can see that you are been taken seriously.   *Professional perspective:*   - It is helpful to establish rapport when beginning therapy or during the first encounter, find out where the patient is and start helping from there. - Rapport is the foundation for communication; fosters open discussion, prevents misunderstanding, facilitate further exploration of barriers to full participation, negotiating rehab programs. It can be developed by learning what patients value. - Helping involves ‘ownership in helping’; a sense of personal ownership of what happens to the patient, ‘getting patients to see’; getting patient to acknowledge and value their progress even when gains are minimal or not apparent, and ‘being there’; providing comfort and support, attesting to and sharing in patient’s suffering | Cooper et al (50s)  Palmadottir (78s)  Peiris et al (80s), Payton & Nelson (99s)  Waters et al (93s)  Crepeau and Garren (52s),  Gyllensten et al (61s)  Harman et al (63s)  Rosa et al (84s) |
|  | Empathy | *Patient perspective:*   - Empathetic interactions involve therapists having sufficient time, was calm and friendly, showed interest in the patient and took patient’s reactions seriously. - Empathy involves locating the patient at the centre of the therapeutic encounter, and make them feel understood and respected. - Empathy means sensitivity to patients needs and listening to concerns and dealing with us in a ‘respectful’ and ‘sympathetic way’. - Interpersonal aspects of clinical interaction such as caring behavior is closely associated with caring behaviour during clinical assessment. Saying ‘its nice if you have a small’ pain and ‘it’s hurting’ felt like it didn’t matter. - Supportive professionals show empathy; the ability to truly understand what it feels like to live with a disease.   *Professional perspective:*   - It was important to have ‘compassion’, ‘fairness’, and ‘empathy’ towards patient’s colleagues and students. - Signifies therapist’s ability to listen actively, be attentive and understand the message the patient wants to express - This involves showing concern for patient’s comfort, indicating understanding, accepting feeling, offering feeling, offering self and showing solidarity. - Its important to take time to show compassion and be quite personable such as saying hello, sit down | Ekerholt & Bergland (55s)  Kidd et al (68s),  May (74s)  Waters et a (93s)  Schoster et al (100s)  Aguilar et al (44s)  Harman et al (63s), Norby et al (75s)  Owen & Good (77s)  Waters et al (93s) |
|  | Caring | *Patient perspective:*   - Attention reflects caring; watchfulness and talking ‘enough’ are sufficient to feel ‘constant’ attention - A caring attitude of paying attention to the person’s feelings and showing interest in his or her life situation is necessary for the relationship to be experienced as positive.   *Professional perspective:*   - Caring involves focusing on the connection to patients as fellow human beings and the illness experience; embracing the emotional and physical well-being of patients; reciprocity of affection and care from patients; essential hands-on therapy; partnering with patients for empowerment; receptivity to patients, willingness to displace own judgments and prejudices; understanding patient motivations; balancing emotional, psychological and physical support; creativity, faithfulness to one’s moral foundations and focus on full significance of the situation. | Crepeau and Garren (52s)  Palmadottir (78s)  Greenfield et al (60s) |
|  | Interest/Concern | *Patient perspective:*   - Patient appreciate personalized and genuine interest in their health problem and typified by sensing therapists understand or connect with their personal circumstances. - Requires therapists to be warm, considerate and attentive to patient needs as opposed to little contact and a lack of closeness. - It was ‘lovely, really refreshing to be treated like a human being and shows the therapist is interested in your physical and mental well-bieng.   *Professional perspective:*   - It is important to show interest in patients. | Hinman et al (65s)  Palmadottir (78s)  Waters et al (93s)  Gard et al (58s) |
|  | Faith in therapist | *Patient perspective:*   - Lack of faith in the therapist leads to clients ignoring advice and failure to adhere to home exercises   *Professional perspective:* | Liddle et al (71s) |
|  | Friendliness/  Courtesy | *Patient perspective:*   - A friendly attitude puts patients at ease, allows relaxation and shows helpfulness. - Interactions that are relaxed like a friendship and based on feelings of worth, dignity and warmth are perceived as a fellowship. - Friendly therapists alleviate boredom - A good therapist is friendly, supportive, considerate, patient, and genuine   *Professional perspective:*   - Despite differences, strong alliances were built through attentive reflective judgments exercises by therapists. | May (74s)  Palmadottir (78s)  Peiris et al (80s)  Potter et al (83s)  Morrison (98s) |
|  | Warmth | *Patient perspective:*   - Extended and frequent contact on an individual basis. - A good therapist has a positive disposition, non-judgemental, enjoys the job, not egotistical, puts the patient at ease during examination and treatment and creates a pleasant and welcoming environment. - Patients are grateful for being so well received and for the fact that their demands were not so high. - The openness and warmth of the therapeutic encounter transcends any conflict and facilitates continued engagement in the presence of skepticism or concern. - Therapists contribute to the development of the relationship by being enjoyable, staying positive in their outlook and evoking warmth – ‘not treating me like an idiot’   *Professional perspective:*   - An important aspect of treatment outcome is to provide a positive and relaxed interpersonal climate to make the patient feel safe and secure in treatment sessions. | Palmadottir (78s)  Potter et al (83s)  Wikman & Faltholm (92s)  Wilson et l (94s)  Morrison (98s)  Norby et al (75s) |
| **Expectation** | Therapy and Outcomes | *Patient perspective:*   - Patients expected recommendations on ‘sort of things that could be done to help’ with recovery. - Therapists need to be “sensitive to frustrations and concerns about therapy expectations”. - Treatment expectations include individual advice and exercise, supervision, follow-up on progress and a proactive approach. - Lack of an early and appreciable response to therapy, expecting a therapist-led and ‘hands-on’ intervention hinders progress   *Professional perspective:*   - Professional’s view of their roles that aligns with a hand-on approach limits outcome. - Patients with unrealistic expectations of the therapist and/or treatment, preconceived ideas about therapy, multiple problems that cause high pain or demand extra management time, low pain threshold, chronic pain, pain-focused can be challenging. - Difficulties arise when expectations of consultations are not fulfilled. - Unmet expectations result from patient dissonance | Bassett and Tango (46s).  Ekerholt & Bergland (55s)  Liddle et al (71s)  Littlewood et al (72s)  Littlewood et al (72s)  Potter et al (82s)  Waters et al (93s) |
| **Individualized care** | Holistic approach | *Patient perspective:*   - Each participant story described the importance of ‘respecting the person, cultural sensitivity, and sense of comfort during care’. - Holistic understanding of the client’s conditions enhances the ability to work collaboratively to resolve occupational performance issues.   *Professional perspective:*   - Respect encompasses accepting patients’ decisions even with disagreement on therapy plans. - Understanding the context of the whole person includes patient’s cultures, backgrounds, values, rights, experiences, personal journeys and personal space is important. | Bassett and Tango (46s)  Morrison (98s)  Aguilar et al (44s)  Crepeau & Garren (52s), Gyllensten et al (61s) |
|  | Responsiveness | *Patient perspective:*   - Individual needs are addressed when ‘exercises make sense and are well explained’ and perceived as being met in group settings when therapists lead the group. - A good therapist puts patients needs first, treats each patient as an individual and appreciates differences between people e.g. physical culture   *Professional perspective:*   - It is important to develop sensitivity and use one’s intuition. ‘It is about living in the here and now’. - Diversity in patient population can create an unintentional communication gap.   *Observer perspective:*   - Clients distinctive characteristics, prior experiences, and/or health conditions might translate into some clients being unable or unwilling to engage actively in a collaborative therapeutic endeavour. | Cooper et al (50s)  Potter et al (83s)  Gyllensten et al (61s)  Karnad & McLean (67s)  Morrison (98s) |
| **Influencing factors** | External factors | *Patient perspective:*   - Organizational issues such as quick access; flexible appointment system, less than 5-10 minutes wait times, having enough time and feeling rushed and protracted referral system; being able to see therapist in the face of a flare up improves satisfaction. - Environmental factors such as duration of therapy attendance is affected by excessive patient/therapist ratio, delay in schedule, variable number of patients across days; interruptions in service delivery, waiting times in sequence of treatment, patient safety and the physical environment; facility design, ambient conditions and social factors can affect service quality and the client-therapist relationship. - A good therapist is clean and hygienic, is flexible and creates ease of access for the injured or disabled person. - Patient organizations can provide a feeling of not being alone with a problem through help and advice.   *Professional perspective:*   - Use of a horse-shoe table to treat multiple patients increase motivation and information sharing. - The underlying culture of a clinic; staff dynamics, working together, positive clinic atmosphere, affects the caring attitudes, beliefs, and behaviors of impressionable therapists. - External prerequisite includes teamwork, available time and services, physical work environment. - Privacy and confidentiality; lack of privacy, space, inadequate and enforced proximity, lack of soundproofing, physical environment; differential familiarity and positioning and formal regulations; rules, conduct, guidelines, autonomy and rights affect the power base - Patient load and work restrictions are barriers to open communication. - Providing patient with much time during the first encounter is a manifestation of interest in the patient. - Low clinic efficiency causing extended waiting times leads to more perceived health issues and feelings of disrespect from patients.   *Observer perspective*   - Individual’s in the client’s environment can have an impact on the rehabilitation process such as limited confidentiality. | Cooper et al (50s)  May (74s)  Medina-Mirapiex et al (75s)  Potter et al (83s)  Wikman & Faltholm (92s)  Crepeau & Garren (52s)  Greenfield et al (59s) (60s)  Gyllensten et al (61s)  Harrison & Williams (62s)  Karnad & McLean (67s),  Mann & Gooberman-Hill (73s)  Kumlin &Kroksmark (69s)  Waters et al (93s)  Morrison (98s) |
|  | Therapist prerequisite | *Patient perspective:*   - A competent therapist ‘know what they are talking about’ and have a ‘great depth of knowledge’. - Therapists technical expertise impacts perception of service quality and considered as important as the treatment content - Care provider’s style including how clinical knowledge is provided, feedback during exercise instruction, giving reminders and monitoring results and engagement have a positive or negative influence on engagement. - Patients want a therapist that is confident their use of knowledge and expertise, their self-confidence and ability to create confidence in the patient. - Therapist self-knowledge; own reflections on values, norms, feelings, resources and limitations, influences the helping process. - Lack of caring and basic trust is evidenced by therapists appearing in the role of a superior, looking down on the client and not listening or paying attention can lead to withdrawal and poor attendance at sessions. - Adopting a ‘vulnerable and fallible’ approach compared to a’ knowledgeable expert’ role creates a lighter clinical atmosphere where experimentation and ‘imperfect’ efforts at exercise.   *Professional perspective:*   - Humor is a form of bantering rapport and a central component of how to engage patients. - It is important to admit your limitations (knowledge), ask for help when needed and be comfortable with patient criticism. - Internal prerequisites valued include practical professional skills and patient experience, theoretical professional development, negative and/or positive life experiences, personality typified by humor, cheerfulness, and flexibility. - Reflections and valuations of life experiences like personal crisis can facilitate the helping process. - Adapting to the patient means the therapist is flexible to the different personalities of the patient’s behaviors, needs, emotional and functional levels. - Learning to seek assistance from other therapists through team meetings can assist with dealing with difficult encounters. - Be honest and encourage honesty. | Cooper et al (50s)  Del Bano-Aledo et al (54s), Hurley et al (66s)  Escolar-Reina et al (56s)  Kidd et al (68s)  Norby et al (76s)  Palmadottir (78s)  Wilson et al (94s)  Crepeau & Garren (52s)  Gard et al (58s)  Gyllensten et al (61s)  Norby et al (76s)  Norby et al (76s)  Petursdottir et al (81s)  Potter et al (82s)  Potter et al (82s) |
|  | Patient prerequisite | *Patient perspective:*   - Personal attributes such as being driven, creating space for exercise, creating a personal routine and recognizing the role of partners stimulate engagement. - Personality traits desired include adaptability, taking initiative, being positive, cheerful and not lingering on negative circumstances. - Ability to learn from mistakes and experiences. - The ability to work with both the emotion and response to emotional distress strengthens the alliance and allows for continued progress.   *Professional perspective:*   - Patients who are very ‘positive about life’, ‘quite outgoing’, ‘very confident in themselves’ and ‘quite determined’ are often successful in therapy. - Patients who are passive, dependent, angry, aggressive, or think “they know it all”, avoid taking responsibility, dependence on a specific treatment and/or therapist, manipulative, generalise bad experiences to all situations, need to be chased up, deny their problems, have a performance drive that is too high and think negatively are very hard to deal with in private PT practice.   *Observer perspective:*   - Cooperative patients go along with what therapists propose for goals and who were willing to do what therapists asked of them. | Littlewood et al (72s)  Littlewood et al (72s)  Wikman & Faltholm (92s)  Wilson et al (94s)  Palmadottir et al (78s)  Potter et al (82s)  Rosa & Hasselkus (85s) |
| **Partnership** | Mutual Understanding | *Patient perspective:*   - There is a sense of reciprocity and obligation that patients feel towards therapists; particularly the desire to not let clinicians down. - Understanding from the professional is very important   *Professional perspective:*   - It is important to ‘understand the individual patient and their interests, goals, thoughts and values…adapt treatment to meet changing needs’. - To effectively participate in decision making, patients need an understanding of the problem and the benefits and limitations associated with the treatment option. - It is important to seek for common ground of mutual understanding; exploring connections between tasks and goals, being sensitive to the patient’s needs for readjustment and checking if verbal and nonverbal messages are interpreted appropriately. - Feeling locked in ambivalence and uncertainty can be resolved with internal dialogues, sharing experience with the patient or asking for advice.   *Observer perspective:*   - Therapists must deal with emotional discomfort in order to create a milieu conducive to client development. | Campbell et al (48s)  Petursdottir et al (81s)  Aguilar et al (44s)  Stenner et al (85s)  Øien ()  Øien ()  Morrison (98s) |
|  | Respect | *Patient perspective:*   - Interpersonal manners include friendly and respectful behavior, emotional support and sensitivity to the patient’s status impacts service quality. - Therapists should be sensitive and create a safe environment, not cross patient boundaries especially in vulnerable situations. - Clients value leading principle where their choices as respected as long as safety is not at risk. - It is important to be taken seriously, to ‘be confirmed’ and ‘not be being insane’. - It’s an act of respect if someone introduces themselves to you and not talking about me but talking to me.   *Professional perspectives:*   - Therapists admired therapists who put the client at the centre of everything that they’re doing and perceived patients as their motivation for practicing. - Helping process based on respect for the patient’s suggestions, comments and choices leads to the strategy “doing with the patient’ rather than doing for him or her. | Del Bano-Aledo et al (54s)  Ekerholt & Bergland (55s)  Palmadottir (78s)  Wikman & Faltholm (92s)  Waters et al (93s)  Aguilar et al (44s)  Norby et al (75s) |
|  | Trust | *Patient perspective:*   - Establishing a trusting relationship creates a comfortable opportunity and atmosphere to air concerns and doubts and promote faith in therapist’s interest in the patient. ”… just must have that communication, that comfortable atmosphere has to be there”. - Trust assists with continued engagement despite challenges to confidence, lack of immediate results and despite increased program difficulty. - Informality of interactions can create a trusting relationship. - Trusting the therapist to choose how much therapy is needed reflects patients do not associate amount of therapy with their progress. - Trust reflects vulnerability regarding a lack of knowledge or alternatives to solve health issues and can be assumed by association to the role of the professional or the institution.   *Professional perspectives:*   - It is important that patients trust the therapist’s decision “the issue of trust is an absolute requirement in a clinical setting to ensure that patients know that what they tell you will be kept in confidence” - Therapist have a strong interest in maintaining trust in their profession in line with professional roles. - Feeling rushed during appointments impedes the development of trust. - Obtaining informed consent is part of the trust building process | Bunzli et al (47s)  Harman et al (63s)  Palmadottir (78s)  Peiris et al (80s)  Aguilar et al (44s)  Waters et al (93s)  Larsson et a (70s)  Potter et al (82s)  Waters et al (93s)  Waters et al (93s) |
|  | Knowledge Exchange | *Patient perspectives:*   - It is important to collaborate on issues of importance such as discharge planning, goal setting, and decision making. - A trusting and collaborative relationship is built with care, support and guidance. - Information shared should cover aspects of the problem, role in treatment, treatment process and prognosis - A good therapist gives clear explanations about the problem and treatment at the appropriate level.   *Professional perspectives*:   - This involves engaging the client in interaction…understanding the patient, seeing what they want…negotiating to find common ground…supporting patients to find the means to achieve goals”. - It is important to be jointly responsible for the outcome; deciding goals from client’s view, work together using the patient’s driving force as motivation, create a framework of participation built on client’s interest, therapist capability to explain illness and disability affect client’s feelings, ideas and expectations. - Information sharing should be mutual…” directed towards advice, spontaneous answers and reactions to the information that the patient provides. - Providing explanations using analogies and case histories making sure to put information in simple terms. - Shared decision making involves clinician providing information to the patient on the management options in an unbiased way. - Persuading patients to think differently requires propositional knowledge can be described as “collaboration where the professional’s knowledge informs decision making”   *Observer perspective*:   - This requires diverse strategies such as direct questions; broad, open-ended questions requiring specific information or direct answers from patients or counseling; answering patient’s questions, building on patient’s questions and ideas, encouraging patients to continue, encouraging comparison and description, reflecting, paraphrasing, restating, summarizing to open up, clarifying patient’s ideas, exploring, using silence and offering observation. - Use of “chain of evidence and feedback” is used to break causal link by challenging patients to look at the evidence of their perceptions an consider changing their view if the evidence did not add up. | Aguilar et al (44s)  Cipriani et al (49s)  May (74s)  Potter et al (83s)  Hurley et al (66s)  Bassett and Tango (46s)  Larsson et al (70s)  Kumlin & Kroksmark (69s)  Potter et al (82s)  Stenner et al (87s)  Thomson (89s)  Owen & Goode (77s)  Thomson (89s) |
|  | Active Involvement | *Patient perspective:*   - Patients prefer an individualised, communicative decision-making approach. - Self motivation increases with accountability and working together; giving and receiving information and being monitored. - Active input into the treatment process reduces focus on “quick fixes’ and understanding recovery takes time in an ongoing process. - Direction means suggesting activities for therapy should be balanced with opportunities to oppose and urging patient to participate in decision making. - Contributing actively takes time and develops by sharing vulnerable experiences, receive explanations from and getting to know the therapist. - A sensitive balance between confrontation and support is required to ensure patients don’t withdraw in the clinical encounter. - Strong sense of trust combined with confidence to work at own pace and modify activities lead to engaging in recommended tasks independently and comfortably.   *Professional perspective:*   - It is crucial to demand some activity from patients. - Use of technology allows both patients and professionals to be involved in treatment. - Use of therapist-patient contract can be used to make clients active in the process. - Creating a climate of cooperation in which the patient could take an active part.   *Observer perspective:*   - Getting patients to do the work of therapy evokes a feeling of obligation to “cover all the bases”, to “make sure” that patients do what they were “supposed to do” during the encounter. - Backing off from ‘pushing’ patients is a way of accommodating patient’s wishes, providing emotional support, or responding to a sense that patients could not tolerate further physical activity and needed to rest. | Cipriani et al (49s)  Hinman et al (65s)  Liddle et al (71s)  Palmadottir (78s)  Øien ()  Øien ()  Schoster et al (100s)  Gyllensten et al (61s)  Papi et al (79s)  Potter et al (82s)  Thornquist (90s)  Rosa & Hasselkus (85s)  Rosa & Hasselkus (85s) |
|  | Power balance | *Patient perspective:*   - Patients want to be involved in the decision- making process,” knowing what was going on” and do not want to be belittled. Acting aloof, apathetic, demanding, hostile, aggressive, disinterested are negative attributes. Taking responsibility, saying motivated, keenness, being compliant, self-heling, beliefs about locus of control and involvement are positive attributes. - Perception of professional power increases faith in the therapist. - Patients try to give themselves the power to dare to demand; to be treated in the right way or come for therapy at times most suited for them.   *Professional perspective:*   - Professional characteristics such as use of jargon, power of discharge and acting as experts with role status to control assessment, decide and implement treatment regimens are disempowering. Personal characteristics can be positive; honesty, trustworthy, empathy, friendliness, openness, communicative competency, genuine interest or negative: formal, rushed, distracted, threatening, patronising, disinterested. - Therapist should balance client participation and therapist professional responsibility to avoid risks and complications.   *Observer perspective:*   - Patients maintain a sense of control where patients can express their own thoughts and feelings openly in the face of contradiction with the professional. | Harrison & Williams (62s)  Karnad &McLean (67s)  Wikman &Faltholm (92s)  Harrison & Williams (62s)  Larsson et al (70s)  Thomson (89s) |
|  | Working together | *Patient perspective:*   - The therapist is always a part of the total “exercise package” since collaboration is essential. - Experiencing the therapist as a human appears to generate a shared responsibility for treatment.   *Professional perspective:*   - Sense of joining together in mutually supportive partnerships characterized by compatibility, reciprocity, rapport, ‘sharing the work’; carrying part of the load, doing tog and responsibilities of therapy creates a feeling of community and connectedness. - Denotes a sense of feeling on the ‘same wave length’ with patients with regards to coming up with therapy goals, getting patients to work hard, and making progress towards therapy goals.   *Observer perspective:*   - Collaboration mitigates the power balance due to differences in knowledge and social position and encourage clients to become actively involved in problem-solving - Change in therapy is embedded in the effort of seeking for and sharing common ground based on negotiations of initiating; management of the task, participant’s feelings related to the task and the therapeutic relationship and sustaining responsibility. | Swardh et al (88s)  Wilson et al (94s)  Rosa et al (84s)  Rosa & Hasselkus (85s)  Morrison (98s) |
| **Roles and responsibilities** | Professional manner | *Patient perspective:*   - Perceived skills that inspire confidence such as feeling positive about professional’s knowledge base through information giving, thoroughness and manual skills. - A good therapist is punctual, reliable, dedicated, puts patient needs first, maintains confidentiality and a professional distance. - Through a more open human interaction, the distance or “them and us” divide normally present in medical interactions are lessened   *Professional perspective:*   - It is important to maintain boundaries given the ‘hands-on approach’ and ‘license to touch’ and ‘control over personal emotions” level of patient trust. - Therapeutic role includes “empathetic attitude, straightforward communication, giving patients full attention and treating them with respect” | May (74s)  Potter et al (83s)  Wilson et al (94s)  Aguilar et al (44s)  Gyllensten et al (61s) |
|  | Skill and competence | *Patient perspective:*   - Some participants believe in the healing virtue of the hand. “I believe that warm hands signify a warm and caring heart, which I think is important” - Demonstrating professional thoroughness and seriousness reduces doubt in the therapist skill and competence. - Patents want a relationship that allows space to recognise therapist’s knowledge and have input into treatment plan and decisions. - A good therapist has appropriate skills and knowledge, knows his/her limitations, seeks further knowledge as required to help patients, keeps up-to-date with the patient status. - The means used by therapists to cope with care-related pain includes evoking empathy, collaborative dialogue, seeking feedback, encouragement, motivation and showing understanding. - Perceiving the therapists as an expert and knowledge can result in a positive interaction.   *Professional perspective:*   - Reflection-interaction involves learning from each encounter and use knowledge gained with new patients. - First encounter anxiety and uncertainty with patients can be alleviated by understanding the diagnosis and its implication; searching for medical information or a sense of security by relying on own knowledge, proficiencies and experiences. - Being paternalistic means setting goals from therapist point of view, using the fact that the therapist possesses sound knowledge to inspire confidence, and expect patients to follow directions even when they question decisions. - Professional knowledge, education and experience guide attention to patient behavior that enables the patient to express him/herself. | Bassett and Tango (46s)  Ekerholt & Bergland (55s)  Kidd et al (68s)  Potter et al (83s)  Favre et al (95s)  Freene et al (98s)  Gyllensten et al (61s)  Kumlin & Kroksmark (69s)  Larsson et al (70s)  Norby et al (76s) |
|  | Support | *Patient perspective:*   - Therapist support and encouragement inspires confidence. - Ongoing support and reassurance from the therapist is key to enhance motivation. - Encouragement from the professional is what keeps “me going”. - Support, encouragement and back-up from professionals was most appreciated. - Patients request training, exercises and supportive conversations. - The supportive professional qualities paying personal attention to clients, learning individual names, skillfully demonstrating tasks, preparing answers to questions and competently understanding and suggesting appropriate activities   Professional perspective:   - Successful engagement can be facilitated through on—going support from therapist. - Use of technology could be a measure of clinical treatment that would be objective and evidence based to support analytical practice in the therapeutic encounter. - Connection felt with patients develops from therapist support; presence and nurtured understanding. - The feelings of safety; having someone to turn to and to be connected with are important considerations during therapy. | Bamford and Walker (45s)  Liddle et al (71s), Littlewood et a (72s),  Petursdottir et al (81s)  Wikman & Faltholm (92s)  Schoster et al (100s)  Littlewood et a (72s)  Papi et al (79s)  Rosa et al (84s)  Swardh et al (85s) |
|  | Motivator or  Encourager | *Patient perspective*   - Socialization and encouragement from patients and professionals creates a motivational atmosphere. - Clinical appointments were encouraging and positive and make patients want to exercise.   *Professional perspective*   - Therapists should help patients see things in a more positive and optimistic way. - Key motivational strategies include focusing on what the patient finds interesting and valuable and describing information and explanation of treatment goals; ‘showing a different angle’ or ‘telling patients what’s good for them’. - Technology can motivate patients and along day-to-day surveillance and enhance the client-therapist relationship. - Positive reinforcement, encouragement and rewards motivate patients who are ‘difficult’. - Role of therapist is to motivate; encourage, instruct and observe during therapy sessions. | Peiris et al (80s)  Kingston et al (97s)  Gyllensten et al (61s)  Norby et al (76s)  Papi et al (79s)  Potter et al (82s)  Swardh et al (88s) |
|  | Educator or  Adviser or  Guide | *Patient perspective:*   - The role of adviser or guide aids patients in getting what they want in therapy without limiting the sense of responsibility and power. - A good therapist explains what he/she is doing and why during assessment and treatment.   *Professional perspective:*   - Education involves sharing knowledge with patients, reflection and awareness; helping patients understand the connection between events, symptoms and treatment. - Good teaching skills extends beyond giving information to shifting paradigms.   *Observer perspective:*   - This role includes giving unsolicited information, opinions, interpretations, advice, evaluating, suggesting, citing an authority other than the patient, and relating own experience and ideas | Palmadottir (78s)  Potter et al (83s)  Gyllensten et al (61s)  Thomson (89s)  Owen & Goode (77s) |
|  | Activator of resources | *Patient perspective:*   - Encouraging patient involvement facilitates adherence to exercises and activities. - A sense of coherence develops from receiving and interpreting all types of information and gradually learn to cope with the new situation or from the inside through their own lived experience. - Working with the whole person creates a safe and therapeutic space, where hope is fostered, possibilities emerge, and encourages physical experimentation with functioning in the ‘real world’ outside of the treatment room as patients take on more challenges. - Progress is influenced by support from the therapist and the patient’s ability to influence their own outcome when the therapeutic context invokes a perception of caring, safety and support   *Professional perspective:*   - The therapeutic process should involve identifying and strengthening patient’s resources. - During the first encounter a more successful strategy was to show interest in the patient as an individual, to offer patients opportunities to describe their problems, express their will or intention to changes in health status or treatment direction. - Strengthening of self involves helping patients express themselves, finding some structure, setting limits in daily situations, finding the level of action of the patient and giving feedback. | Veenhof et al (90s)  Wikman & Faltholm (92s)  Waters et al (93s)  Wilson et al (94s)  Gyllensten et al (61s)  Kumlin & Kroksmark (69s)  Norby et al (76s) |
|  | Active follow-up | *Patient perspective:*   - Pro-active follow-up by the therapist is a great motivator for engagement. - A good therapist follows up and actively involves the patient. - Backup and support from the therapist is perceived as convenient, pleasant, and positive are remains desired despite the knowledge, skills, and responsibility for doing exercises independently. - Telephone calls during home-based program were key enablers providing encouragement and advice in an efficient manner - Participants appreciated follow-up to check up on performance of home exercises   *Professional perspective:*   - Following up difficult patients with a phone call enhances the relationship. | Littlewood et al (72s)  Potter et al (83s)  Swardh et al (88s)  Freene et al (98s)  Schoster et al (100s)  Potter et al (82s) |
