# Supplementary material for: Characteristics of therapeutic alliance in musculoskeletal physiotherapy and occupational therapy practice: a scoping review of the literature
Source: BMC Health Serv Res. 2017 May 30;17:375. doi: 10.1186/s12913-017-2311-3 (PMC5450083; doi:10.1186/s12913-017-2311-3)
Supplement: Supplementary file 3 — Perspectives on therapeutic alliance from participants. This file highlights the perspective and experiences of participants on therapeutic alliance. (DOCX 400 kb) [file 12913_2017_2311_MOESM3_ESM.docx]

**Additional file 3:** Perspectives on therapeutic alliance from participants
